# Prediction of prime editing insertion efficiencies using sequence features and DNA repair determinants

**DOI:** 10.1038/s41587-023-01678-y

**Published:** 2023-02-16

**Authors:** Jonas Koeppel, Juliane Weller, Elin Madli Peets, Ananth Pallaseni, Ivan Kuzmin, Uku Raudvere, Hedi Peterson, Fabio Giuseppe Liberante, Leopold Parts

**Affiliations:** 1 Wellcome Sanger Institute, Hinxton, UK; 2 Department of Computer Science, University of Tartu, Tartu, Estonia

**Keywords:** Functional genomics, Synthetic biology, Computational models, Genetic engineering, High-throughput screening

## Abstract

Most short sequences can be precisely written into a selected genomic target using prime editing; however, it remains unclear what factors govern insertion. We design a library of 3,604 sequences of various lengths and measure the frequency of their insertion into four genomic sites in three human cell lines, using different prime editor systems in varying DNA repair contexts. We find that length, nucleotide composition and secondary structure of the insertion sequence all affect insertion rates. We also discover that the 3′ flap nucleases TREX1 and TREX2 suppress the insertion of longer sequences. Combining the sequence and repair features into a machine learning model, we can predict relative frequency of insertions into a site with *R* = 0.70. Finally, we demonstrate how our accurate prediction and user-friendly software help choose codon variants of common fusion tags that insert at high efficiency, and provide a catalog of empirically determined insertion rates for over a hundred useful sequences.

## Abstract (web summary)

Prime editing efficiency is affected by insertion sequence features, secondary structure and DNA repair context.

## Main

The efficient insertion of short DNA sequences into genomes could change the course of biotechnology and medicine^1,2^. Small insertions can encode protein tags for purification and visualization, or manipulate protein function by altering protein localization, half-life or interaction profiles. Integrating sequences for transcription factor binding sites and splicing modulators provides control over gene expression while introducing structural elements or recombinase sites can change DNA conformation and provide a substrate for large-scale engineering. For therapeutic opportunities, over 16,000 small deletion variants have been causally linked to disease^3,4^, and could in principle be restored by inserting the missing sequence^5,6^. A prominent example is cystic fibrosis, where 70% of cases are caused by a three-nucleotide (nt) deletion^7,8^. To enable reversing these mutations in practice, a technology must integrate insertions efficiently, accurately and safely, avoiding the unintended outcomes and double-strand break stress that hampers existing Cas9-based therapies^9–11^.

Prime editors can insert short DNA sequences without generating double-strand breaks or requiring an external template. They consist of a nicking version of Cas9 fused to a reverse transcriptase domain, which is complexed with a prime editing guide RNA (pegRNA)^12^. The pegRNA comprises a primer binding site homologous to the sequence in the target, and a reverse transcriptase template that includes the intended edit, all in the 3′ extension of a standard CRISPR–Cas9 guide RNA. At the target site, Cas9 nicks one strand of the genomic DNA, which then anneals to the primer binding site on the pegRNA, and is extended by the Cas9-fused reverse transcriptase using the pegRNA-encoded template sequence. Next, DNA repair mechanisms resolve the conflicting sequences on the two DNA strands, ultimately writing the intended edit into the genome. Where CRISPR–Cas9 was compared with molecular scissors capable of disrupting target genes, and base editors were seen as molecular pencils for their ability to substitute single nucleotides, prime editors can be described as molecular word processors, able to perform search and replace operations directly on the genome^13–16^.

The prime editing system is complex, and the determinants of its efficiency are not fully understood. Several partly independent steps, including three DNA binding events and successful DNA repair, are needed to produce an edit, each potentially influenced by the introduced sequence. In the largest study so far to understand these biases, Kim et al. comprehensively tested the consequences of varying the reverse transcription templates and primer binding site lengths using a library of 55,000 pegRNAs. The editing rate increased with Cas9 guide RNA activity, as well as GC content and melting temperature of the primer binding site. While further optimization of sequences was possible, primer binding sites of 11–13 nt and reverse transcriptase templates of 10–12 nt had the highest average editing efficiencies^17^.

The majority of libraries used by Kim et al. contained the same single-nucleotide substitution 5 nt upstream of the nick site. Similarly, nearly all investigations of prime editing efficacy to date have predominantly focused on single-nucleotide substitutions^12,17–21^. Of the many possible useful sequences in molecular biology, only a handful have been introduced with prime editing^12^. Therefore, in contrast to a relatively deep understanding of Cas9 mutagenesis^10,22–24^ and base editing outcomes^25–27^, very little is known about how the inserted sequence affects efficiency, and the length range of insertions feasible by prime editing has not been defined.

Here, we systematically measure the insertion efficiency of 3,604 sequences in several target sites and a variety of cellular and repair pathway contexts. We find that insertion sequence length, nucleotide composition and secondary structure all affect insertion efficiency. Moreover, we define the precise effect of mismatch repair (MMR) on thousands of insertion sequences and discover that overexpression of the 3′ flap nucleases TREX1 and TREX2 abolished the insertion of longer sequences. Together, sequence features and repair pathway activity explain most of the variation in insertion rate. We then use these insights to train a sequence-based prediction model informed by MMR efficiency that predicts editing outcomes for novel sequences with high accuracy and demonstrate the model’s usefulness for the selection of optimal reagents for new insertions.

## Results

**Fig. 1:**
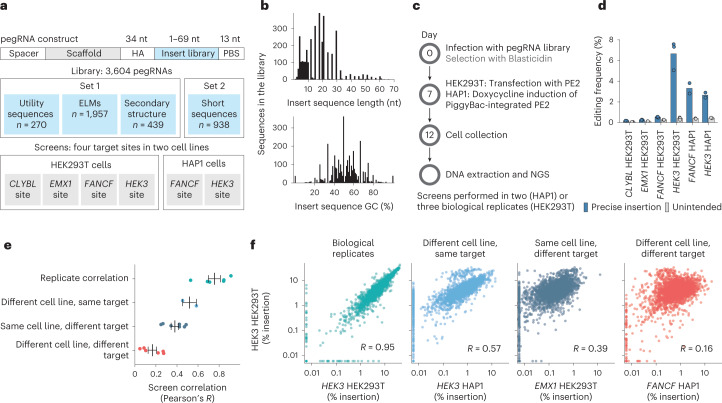
High-throughput measurement of prime insertion efficiencies. **a**, Screen setup. Set 1 and Set 2 libraries were screened separately and data merged (Methods); panels **d**–**f** reflect Set 1 results only. **b**, Library composition. The number of sequences in the library (*y* axis) with different insert sequence lengths (*x* axis, top panel) and %GC content (*x* axis, bottom panel). **c**, Experimental design. NGS, next generation sequencing. **d**, Editing frequencies. Average mutation frequency (*y* axis) for different screens (*x* axis) stratified by mutation type (blue, insertions; gray, unintended outcomes). Markers represent one replicate and bars the average across *n* = 3 biological replicates. **e**, Replicate concordance. Pearson’s *R* between insertion rates in two screens (*x* axis) for different comparisons (*y* axis, colors). Markers, correlation value of one pair of screens (for replicate correlations, mean of pairwise comparison across *n* = 3 biological replicates); line and whiskers, mean and s.e.m. **f**, Representative examples of categories from **e**. Percentage insertion in the *HEK3* locus in HEK293T cells (*y* axis) compared with values (*x* axis) in other contexts (panels, colors) for insertion sequences (markers). Left panel, comparison of biological replicates; other panels, comparison of replicate averages. Label, *R* of values in linear scale. Colors as in **e**.

We sought to systematically characterize how the length and composition of inserted sequence, as well as cell line, target site and the version of the prime editor system, affect insertion rates. To do so, we designed 3,604 pegRNAs encoding insertions immediately upstream of the nick site. These comprise 270 sequences useful for molecular biology (for example, His-6 tag, recombinase sites and mNeonGreen-11 (ref. ^28^)); 1,957 eukaryotic linear motifs^29–31^; 439 sequences with variable secondary structure; all single nucleotides, dinucleotides, trinucleotides and tetranucleotides; and 100 random sequences of each length between 5 and 10 nt (Fig. 1a). Insertions ranged from the length of 1 to 69 nt, and varied in GC content (Fig. 1b), while the primer binding site and homology arm lengths in the pegRNA were fixed to 13 and 34 nt, respectively. We used lentiviruses to deliver the libraries against four target sites (three previously tested: *HEK3*, *EMX1*, *FANCF*^12^, and the safe-harbor *CLYBL* locus^32^) in two cell lines (HEK293T and HAP1), followed by transient transfection of the prime editor 2 plasmid (HEK293T cells) or doxycycline induction of PiggyBac transposase integrated prime editor (HAP1 cells), five d of selection and sequencing of two amplicons from the cell pool, one of the targeted locus and one of the pegRNA locus (Fig. 1c). We calculated insertion efficiencies as the fraction of reads in the target site amplicon with a given insertion divided by the fraction of reads for the pegRNA encoding it in the pegRNA amplicon, and analyzed them as the main statistic in the rest of the study.

Insertion efficiencies of sequences varied widely. The top 5% of templates were inserted 27–134 times more efficiently than the bottom 5% across the various target site and cell line combinations (Supplementary Fig. 1a,b), indicating substantial sequence-dependent variation. The insertion rates were highly consistent across biological replicates (median *R* = 0.70; Supplementary Fig. 1c–i), but differed in magnitude across screens (average across pegRNAs, 0.18% for the *CLYBL* locus in HEK293T to 6.7% for the *HEK3* locus in HEK293T cells; Fig. 1d). Unintended editing outcomes we observed included single-base mutations, small insertions and deletions around the nicking site, deletions overlapping primer binding site and reverse transcription template, insertion of mutated library sequences, duplications of the reverse transcription template, as well as partial scaffold integrations (Fig. 1d and Supplementary Fig. 2a–c). These outcomes were rare overall (0.06–0.45%). Base changes at the target site were infrequent in reads with and without insertions (0.038% versus 0.030%), but slightly elevated upon insertion immediately downstream of the nick site and for the first nucleotides after the end of the homology arm (Supplementary Fig. 2d–f). Overall, the intended insertions were the dominant mutations generated, and we do not consider the unintended edits further.

To understand the consistency of insertion efficiencies across contexts, we next compared them between replicates, cell lines and target sites. Insertion rates into the same target site in different cell lines were more correlated (mean *R* = 0.52) than into different target sites in the same line (mean *R* = 0.38). The correlation was weakest when both the target site and cell line were different (mean *R* = 0.17; Fig. 1e,f), demonstrating both target sequence-specific and cell line-dependent biases on insertion.

### Insert size and MMR activity effects

**Fig. 2:**
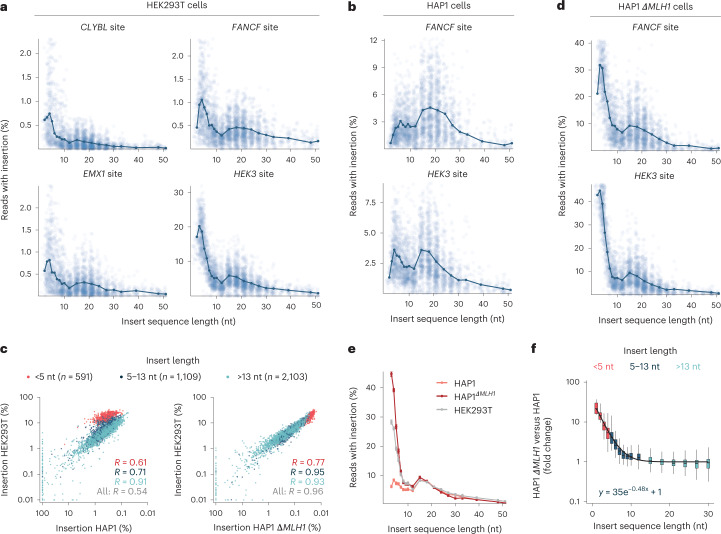
Prime insertion efficiency depends on insert length and MMR. **a**, Insertion rate in HEK293T cells. Percentage of reads with insertion (*y* axis, cut-off at 3 s.d. above mean) for different insert sizes (*x* axis) of individual sequences (blue markers) and averages for lengths with at least 30 measured sequences (dark blue line and markers) at different target sites (panels). Data represent the average of *n* = 3 biological replicates. **b**, As **a**, but for HAP1 cells. **c**, As **a**, but for HAP1 *∆MLH1* cells. **d**, Insertion rate in one cell context (*y* axis) compared with in another context (*x* axis) at the *HEK3* target of individual sequences (markers), comparing HEK293T with HAP1 cells (left panel) and HEK293T cells with HAP1 *∆MLH1* cells (middle panel). Red, short sequences (up to 4 nt); blue, medium sequences (5–13 nt); teal, longer sequences (>13 nt). Label, *R* between rates. The data are an average from *n* = 3 biological replicates (HEK293T) or *n* = 2 biological replicates (HAP1). **e**, Average insertion rates (*y* axis) across insert lengths (*x* axis) with at least 30 measured sequences in various cell line contexts (colors). Data are presented as mean ± s.e.m. *n* = 3 biological replicates (HEK293T) or *n* = 2 biological replicates (HAP1). **f**, The ratio of relative insertion rates (Methods) at the *HEK3* locus between HAP1 *∆MLH1* and HAP1 cells (*y* axis) for different lengths (*x* axis) stratified by colors as in **d**. Box, median and quartiles; whiskers, least extreme of 1.5 times the interquartile range from the quartile and most extreme values. Line, fit from an exponential model (ratio ≈ *a* × exp(−*b* × length) + 1). *n* = 2 biological replicates.

Given the repeatable sequence-dependent variation in insertion rates that spans over three orders of magnitude, we sought to understand the responsible features, starting with insert length. Insertion frequency did not decrease monotonically with insert length in HEK293T cells, but instead, had two modes of high values. First, sequences of 3 and 4 nt were inserted on average 2.0–4.1 times more efficiently than others across the four targeted sites (Fig. 2a). Second, sequences between 15 and 21 nt were inserted on average 1.3–1.6 times more efficiently than 10–14-nt ones, and 1.5–2.0 times more efficiently than sequences longer than 21 nt (Fig. 2a). These relative biases in efficiency were shared between all target sites, despite a 20-fold range of their average insertion rates. Inserts longer than 45 nt were incorporated less frequently, at a screen average rate that is 4–8 times lower than that of sequences shorter than 45 nt. The longest sequence that was inserted at >1% frequency (1.4%, *HEK3* site in HEK293T cells) was 66 nt, demonstrating that integration of moderately long sequences is feasible with prime editing.

In contrast to HEK293T cells, the insertion frequency of the short 1–4-nt sequences was not substantially higher than that of longer ones in HAP1 cells (0.60–1.27 times; Fig. 2b). This reduced the concordance of insertion rates in the two cell lines at the same site (*R* = 0.41 for *FANCF* and 0.54 for *HEK3*; Fig. 2d and Supplementary Fig. 3a) compared with replicates (median *R* = 0.78; Fig. 1e). One possible explanation is MMR proficiency, since HEK293T cells are partly MMR deficient due to promoter methylation of *MLH1* (ref. ^33^), while HAP1 cells are not. The MMR pathway recognizes and excises short mismatches of less than 13 nt and could therefore remove short insertions in HAP1 cells before the nicked strand is re-ligated^34^. Indeed, MMR antagonizes prime editing for substitutions and short insertions^20,35^. Consistent with this explanation, we observed strong correlations between insertion rates in HAP1 and HEK293 cells for sequences longer than 13 nt that are not affected by MMR (*R* = 0.78 for the *FANCF* locus and 0.91 for the *HEK3* locus; Fig. 2c and Supplementary Fig. 3a).

To experimentally test the hypothesis that rates of insertion of short sequences differ between cell lines due to MMR activity, we screened the *HEK3*- and *FANCF*-targeted libraries in HAP1 cells that are knocked out for *MLH1* (HAP1 *∆MLH1*; Fig. 2d and Supplementary Fig. 3b,c). We found that the average insertion rates of 1–4-nt sequences were most affected by the knockout, increasing by 7.2–11-fold, while the rates of 5–13-nt sequences increased 2.1–2.7-fold (Fig. 2e and Supplementary Fig. 3d). Overall, 66% (*HEK3*) and 67% (*FANCF*) of the variance in the fold changes (Fig. 2f and Supplementary Fig. 3d) was explained by a model where the loss of MMR increases the insertion rate of 1-nt sequences by 23–28-fold, with the increase in insertion efficiency dropping 40–48% for every additional nucleotide. The low correlations of insertion rates between HEK293T and wild-type HAP1 cells (*R* = 0.41–0.54) also improved to close to replicate concordance when matching MMR status (*R* = 0.73–0.96 between HEK293T and HAP1 *∆MLH1* cell lines; Fig. 2c and Supplementary Fig. 3a,e). In summary, our findings highlight that MMR proficiency is the major source of independent variation between the tested cellular contexts for prime insertion of short sequences.

### Effects of prime editing steps

**Fig. 3:**
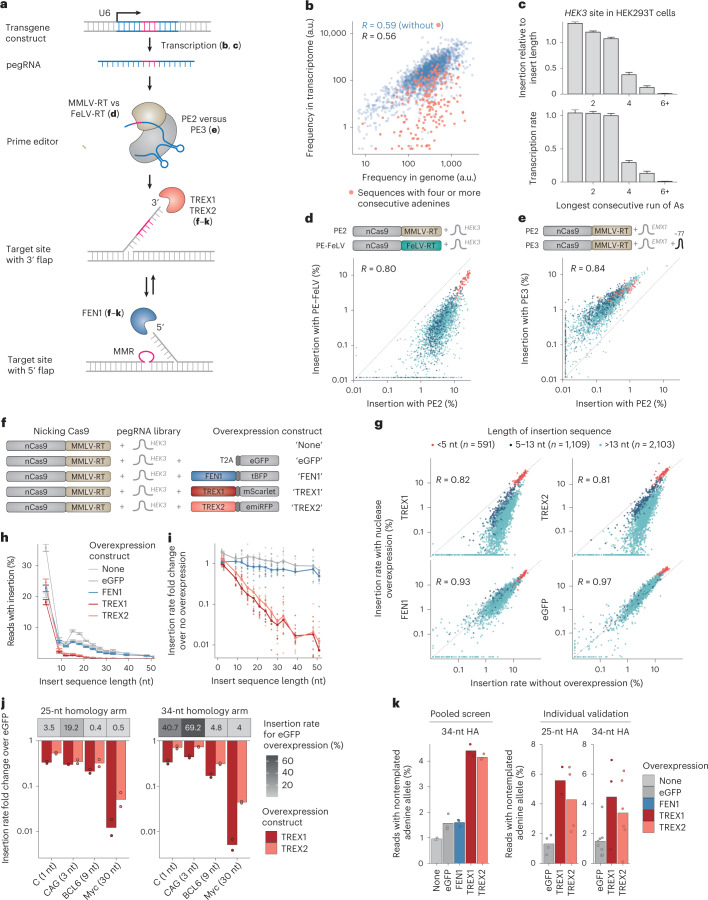
Effects of prime editing steps. **a**, Schematic of molecular steps involved in prime editing. **b**, Normalized pegRNA count derived from sequencing of PCR amplicons from genomic DNA (*x* axis) or PCR amplicons from RNA (*y* axis) for the *HEK3* site in HEK293T cells for individual pegRNAs (markers). Pink, inserts with four or more consecutive adenines. Data represent the average of *n* = 3 biological replicates. **c**, Top panel, average insertion rate relative to length bin median (*y* axis) for inserts stratified by the longest consecutive run of adenines (*x* axis). Bottom panel, instead showing transcription rate (read counts from RNA/read counts from DNA) on the *y* axis. Data are presented as mean ± s.e.m. *n* = 3 biological replicates. **d**, Insertion frequencies at the *HEK3* site in HEK293T using the standard MMLV reverse transcriptase (PE2, *x* axis) and the FeLV reverse transcriptase (PE-FeLV, *y* axis) for different insertion sequences (markers). Colors, number of neighboring points. *n* = 3 biological replicates. **e**, As **d**, but comparing PE3 and PE2 at the *EMX1* site. **f**, Schematic of screens with overexpression constructs. **g**, Insertion frequencies for different overexpressions (*y* axis and panels) compared with no overexpression (*x* axis) for three biological replicate screens (markers) stratified by insertion sequence lengths (colors). **h**, Average insertion rates (*y* axis) across insert lengths (*x* axis) with at least 30 measured sequences for overexpression constructs (colors). Data are presented as mean ± s.e.m. *n* = 3 biological replicates. **i**, As **h**, but instead displaying the insertion rate fold changes of screens with overexpressions compared with no overexpression (*y* axis), calculated from the ratio of sums of all sequences (lines) or of ten randomly sampled sequences. **j**, Top, average insertion frequency (grayscale) of four sequences with varying lengths (*x* axis) when overexpressing eGFP stratified by homology arm lengths (panels). Bottom, insertion rate fold changes compared with eGFP (*y* axis) when overexpressing TREX1 and TREX2 (colors). *n* = 2 biological replicates. **k**, Fraction of the nontemplated adenine allele at the +9 position (*y* axis) for cells with overexpression constructs (*x* axis and colors) stratified by experiment and homology arm lengths (panels). Markers show screen averages from three biological replicates for the pooled screen or from separate pegRNAs for the individual validation experiment.

Having confirmed MMR as a length-dependent determinant of insertion efficiency, we next sought to understand how different steps of prime editing affect insertion rates of our library sequences. Specifically, we dissected the contributions of (1) pegRNA expression, (2) reverse transcription by two different reverse transcriptases, (3) presence of a nicking guide and (4) overexpression of 3′ and 5′ flap nucleases (Fig. 3a).

**Fig. 4:**
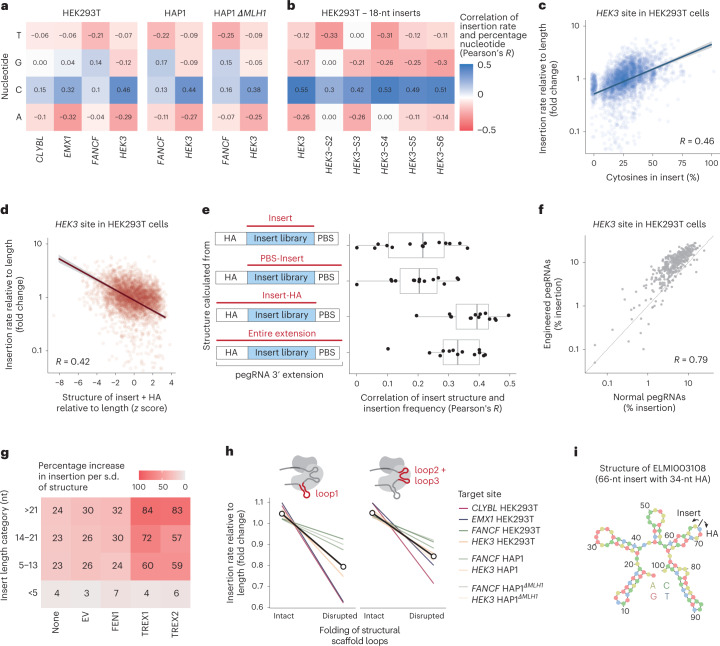
Cytosine content and secondary structure of the insert sequence are positively correlated with the insertion rate. **a**, Correlation of length-normalized insertion rate with nucleotide frequency in the insert (colors) for each nucleotide (*y* axis) in each screen (*x* axis). Data represent the average of *n* = 3 (HEK293T) or *n* = 2 (HAP1) biological replicates. **b**, As **a**, but for a new set of screens with 18-nt inserts and 15-nt homology arms targeting five novel sites within 1 kb of the *HEK3* site. **c**, Insertion rate at the *HEK3* site in HEK293T cells relative to length bin median (*y* axis) for inserts (markers) with different cytosine content (*x* axis). Line, linear regression fit; shaded area, 95% posterior confidence interval of the fit. Data represent the average of *n* = 3 biological replicates. **d**, Insertion rates at the *HEK3* site in HEK293T cells relative to length bin median (*y* axis) for inserts (markers) with calculated Gibbs free energy (∆*G*) from ViennaFold (*x* axis). Line, linear regression fit; shaded area, 95% posterior confidence interval of the fit. Data represent the average of *n* = 3 biological replicates. **e**, Correlation (*x* axis) between insertion rates and insert sequence free energy calculated from different parts of the 3′ extension (*y* axis). Box, median and quartiles; whiskers, least extreme of 1.5 times the interquartile range from the quartile and most extreme values. *n* = 3 (HEK293T) or *n* = 2 (HAP1) biological replicates. **f**, Insertion rates for sequences (markers) at the *HEK3* site in HEK293T for pegRNAs (*x* axis) and epegRNAs (*y* axis). Data represent the average of *n* = 3 biological replicates. **g**, Percentage increase in insertion rate with each standard deviation increase in structure strength (colors) for different overexpression constructs (*x* axis) and insertion sequence lengths (*y* axis). **h**, Insertion rates relative to length bin median (*y* axis) for sequences that disrupt or preserve (*x* axis) scaffold loops (panels). Colored lines show screen medians and the thicker black lines and dots show the median across all screens. **i**, The predicted secondary structure of a 66-nt insert sequence (ELMI003108) with the *HEK3* homology arm.

We first assessed expression levels of pegRNAs targeting the *HEK3* site in HEK293T cells using deep sequencing. Abundance in the transcriptome was well correlated between replicates (median *R* = 0.97; Supplementary Fig. 4a) and with the DNA-derived read count frequency (*R* = 0.56; Fig. 4b). The exceptions were sequences that resulted in four or more consecutive thymines on the pegRNA cassette (adenines in the inserted DNA), which act as transcription terminators for RNA polymerase III (refs. ^36,37^). Upon removing pegRNAs with terminator motifs, the correlation between measured DNA and RNA sequence coverage increased to 0.59 (Fig. 3b). Sequences with four or more consecutive adenines were 4.8-fold less expressed and, accordingly, their average insertion rate was 4.8-fold lower compared with other sequences (Fig. 3c and Supplementary Fig. 4b). Overall, 23 of the 24 inserts (96%) that were not observed in any screen contained at least one run of four or more adenines, highlighting this feature as a useful filter in pegRNA design.

Second, to disentangle the contribution of the reverse transcription step, we made a prime editor construct with the nicking Cas9 fused to an engineered feline leukemia virus reverse transcriptase (MashUp RT: pipettejockey.com) with similar fidelity to the murine leukemia virus one used in prime editor 2. The average insertion rates observed using this construct were 6.7-fold lower compared with the standard PE2 (0.72% and 4.86%, respectively; Supplementary Fig. 5a–d), but highly correlated to PE2 (*R* = 0.80; Fig. 3d). Therefore, the effects of the insert sequence on insertion are not specific to the murine reverse transcriptase used in PE2 and highlight the possibility to perform prime editing experiments with alternative constructs.

The PE3 system includes an additional guide RNA to nick the nonedited strand, which increases editing efficiency as well as indel formation rate^12^. We explored how the addition of this extra sgRNA affects the insertion frequencies of our library. We chose the *EMX1* locus in HEK293T cells where we observed poor insertion efficiencies of 0.28% on average without the nicking guide RNA and cotransfected a nicking guide RNA that targets 77 nt downstream of the pegRNA target^38^. We found that the extra nick increased the average insertion rate by 5.6-fold to 1.5% (Supplementary Fig. 5d–g), and increased the indel rate by 2.3-fold to 0.31%, including deletions between the nick sites of the pegRNA and sgRNA that were not observed for PE2 (Supplementary Fig. 5h). Importantly, the relative insertion rates for sequences in the library were highly concordant between PE2 and PE3 in HEK293T cells (*R* = 0.84; Fig. 4f).

An important step in prime editing is to resolve between the intermediates with a 5′ flap (containing the wild-type sequence) or a 3′ flap (containing the insertion) that compete. We speculated that the activity of the respective flap nucleases can steer the balance between the two outcomes. To test this, we overexpressed the 5′ flap nuclease FEN1 and the 3′ flap nucleases TREX1 and TREX2 in the context of the *HEK3* site-targeting screen in HEK293T cells. As a control, we overexpressed eGFP in the same backbone used for the nucleases (Fig. 3f). The insertion rates after FEN1 or eGFP overexpression were highly correlated to those measured in screens without overexpression (*R* = 0.93 and 0.97; Fig. 3g) with similar length dependence (Fig. 3h and Supplementary Fig. 6a–d). Intriguingly, TREX1 and TREX2 overexpression abolished the insertion of longer sequences. For cells that did not overexpress nucleases or overexpressed eGFP, the average insertion rate for sequences longer than 4 nt was 4.4–6.0% which is 4.4–5.8 times less than for shorter sequences. This is in contrast to cells overexpressing TREX1 and TREX2, where the average insertion rate for sequences >4 nt was only 0.66% or 0.97%, 25.3–26.7-fold lower than that of shorter ones (Fig. 3h,i).

We confirmed that TREX1 and TREX2 antagonize prime insertions in a length-dependent manner. To do so, we cotransfected HEK293T cells with overexpression constructs encoding eGFP, TREX1 or TREX2 (Fig. 3f and Supplementary Fig. 6e) and individual pegRNAs targeting the *HEK3* site encoding a 1-, 3-, 9- or 30-nt insertion (C, CAG, BCL6 binding site and Myc-tag) in the context of 25- or 34-nt homology arms (Fig. 3j). Overexpressing TREX1 and TREX2 decreased editing rates across all insert and homology arm lengths, but disproportionately more for longer inserts (1.6–3.0-fold for the 1-nt insertion compared with 20–108-fold for the 30-nt insertion; Fig. 3k). This effect could be driven by the length of the insert sequence alone or of the entire 3′ flap (corresponding to insertion + homology arm). In line with the results from our pooled screens (Fig. 3i), we observed a strong correlation between the log fold change of insertion rates for TREX1/2 over eGFP and the insert sequence length (*R* = 0.97) which decreased when considering the total extension length (*R* = 0.86–0.92; Supplementary Fig. 6f), suggesting a more important role for the insertion length than the overall flap length.

The *HEK3* locus in HEK293T contains a single-nucleotide variation at position 9 after the prime editor nick site. The pegRNA homology arm encodes a G for this position, while one of the three chromosome copies encodes an A. If a 3′ flap containing the edit and at least 9 nt of the homology arm was fixed into the genome, we would expect a decreased frequency of the A allele. Indeed, for both pooled and validation screen conditions without TREX1/2 overexpression, we only observed 0.95–1.6% (screen averages) of reads with library insertions containing A in the +9 position compared with 33–36% for unedited reads (Fig. 3k). This is in contrast to screens overexpressing TREX1/2 where the percentage of the A allele increased to 3.4–6.9%, suggesting a higher proportion of flaps where the homology arm was digested to below 9 nt (Fig. 3k). Taken together, our data demonstrate that TREX1/2 antagonize the insertion of longer sequences with prime editing, presumably by digesting the 3′ flap intermediate containing the edit.

### Sequence content effects on insertion efficiency

We next examined sequence content-dependent variation in insertion rate. To address this in a length-independent way, we calculated the insertion rate of each insert relative to sequences with the same or similar length (Methods) and then measured its correlation with sequence features, computed from the perspective of the written sequence (that is, the reverse complement of the pegRNA molecule sequence). We observed a consistent cytosine preference across all four target sites and cell lines (Fig. 4a and Supplementary Fig. 7a), with each extra percentage of cytosine in the insert increasing the relative insertion rate by an average of 2.2%. Conversely, the percentages of adenine and thymine decreased insertion rates for all loci and cell lines (Fig. 4a and Supplementary Fig. 7a).

Our observations of nucleotide content effect were limited to four target sites, and moderately variable. To confirm whether the sequence influences hold more broadly, we performed an additional set of screens in HEK293T cells, targeting the original *HEK3* site and five novel sites within 1 kilobase (kb) of the *HEK3* site (dubbed *HEK3-S2* to *HEK3-S6*) with pegRNA libraries encoding 356–388 18-nt inserts on pegRNAs with 15-nt homology arms (average insertion rate 3.2%, median *R* between replicates 0.81; Supplementary Fig. 7b). Reassuringly, the sequence preferences were recapitulated in this experiment, with a strong preference for cytosines (average *R* between insertion rate and cytosine fraction = 0.47; Fig. 4b and Supplementary Fig. 7c).

We next sought to understand how pegRNA secondary structure affects insertion rates. As the strength of the structure depends on the length of the insert, we calculated the secondary structure’s free energy relative to a large sample of sequences of the same length (Methods). We observed that sequences with relatively stronger structures were more efficiently inserted (*R* = 0.46; Fig. 4d). To better understand this effect, we considered which combination of the pegRNA parts (primer binding site, insert and homology arm) gives predicted free energies that best reflect insertion efficacy. We observed the strongest correlation when the structure was calculated from the reverse transcribed portion of the extension (that is, the combination of insert sequence and homology arm; average *R* across screens = 0.38), and the additional inclusion of the primer binding site sequence decreased correlation (Fig. 4e and Supplementary Fig. 8a,b). Further, the free energies of pegRNA extensions designed for one target site always predicted insertion efficiency better at the same site than other target sites (Supplementary Fig. 8c). Since the homology arm is specific to the target, this also explains some of the differences in insertion rates we observed across the target sites.

Structure in the insert and homology arm could increase prime editing efficiency by protecting the pegRNA itself from nuclease degradation, a strategy explored in engineered pegRNAs (epegRNAs) which contain structured RNA elements to the 3′ of the primer binding site^21,39,40^. However, we did not observe an increased abundance of more structured pegRNAs in the transcriptome (Supplementary Fig. 8d), suggesting an alternative mechanism. To better understand the interplay of structure in various parts of the pegRNA and how it affects insertion rates, we screened 439 inserts of varying free energy from the original pegRNA library in the epegRNA construct, targeting the *HEK3* site in HEK293T cells (Supplementary Fig. 8e–g). We found that the additional structure in the insert and homology arm also increased insertion rates for epegRNAs (*R* = −0.34) but to a lesser extent than for regular pegRNAs (*R* = −0.53; Supplementary Fig. 8h), and that the insertion rates between regular and epegRNAs were highly correlated (*R* = 0.79; Fig. 4f). Together, this implies that structure past the protective cap still influences insertion rates via ways beyond transcript abundance, and that our results on insertion efficiencies are relevant for epegRNAs as well.

We further noticed that structure in the reverse transcribed portion of the pegRNA was not correlated to the insertion rates of sequences <5 nt, but was well correlated for longer sequences (Fig. 4g). Since insertion rates of longer sequences are more impacted by overexpression of TREX1 and TREX2, we speculated that the structure protects the reverse transcribed 3′ DNA flap containing the edit from degradation. Indeed, we observed that structure has a 2.4–2.6-fold stronger effect for cells overexpressing TREX1 or TREX2 compared with cells overexpressing FEN1, eGFP or nothing (Fig. 4g and Supplementary Fig. 9a–d).

Structure plays a role in other parts of the pegRNA molecule as well. For instance, the 13 nt of the primer binding site are perfectly complementary to the protospacer (positions 5–17) and can therefore hybridize with each other. If the first nucleotides of the insert create further base pairing with the protospacer and scaffold, the strength of this structure is enhanced, and the protospacer could be sequestered from base pairing with the target site or ribonucleoprotein complex formation with Cas9 could be impaired. To test if this additional pairing affects insertion rates, we predicted minimum free energy configurations of the primer binding site and the first three insert nucleotides with the spacer and the first guanine of the scaffold and observed 27% lower editing rates for inserts with extended base pairing 3 nt into the protospacer compared with no extension (Supplementary Fig. 10a). Finally, we tested if the disruption of the structural scaffold loops, which are required for association with Cas9, by the insert sequence reduces insertion rates. We calculated the minimum free energy configuration of the insert with the scaffold and observed 26% lower average editing for the pegRNAs with the first scaffold loop disrupted (screen range 10–43%) and 20% with the second and third loops (screen range 11–35%) compared with other inserts of the same length (Fig. 4h). This loop dependence is in agreement with recent findings that scaffold variants with additional point mutations to stabilize the stem-loops can increase prime editing efficiencies^41^.

Combining effects of insert sequence length, cytosine content and structure explained why some sequences are inserted much better than others. For example, the long 66-nt ELMI003108 sequence that was inserted in the *HEK3* locus at 1.39% insertion frequency (0.66% on average for the other 10 sequences >66 nt) formed a strong structure together with the *HEK3* homology arm (minimum free energy = −35.2 kcal mol^−1^; 1.5 s.d. lower than the average free energy of 66-nt sequences; Fig. 4i). Other longer sequences that inserted frequently relative to their size were recombinase sites which are often near-palindromic and therefore form strong structures (Supplementary Fig. 10b,c). Finally, our library included eight codon variations of the His-6 tag in forward and reverse orientations. The average insertion difference between the best codon variant and the worst was 13.3-fold, with the highest insertion rate for the cytosine-richest CAC histidine codons (Supplementary Fig. 10d). This directly demonstrates the practical utility of this new understanding for guiding the codon choice for tags to insert (see the Supplementary Note for a more thorough discussion).

### Predicting insertion rates

**Fig. 5:**
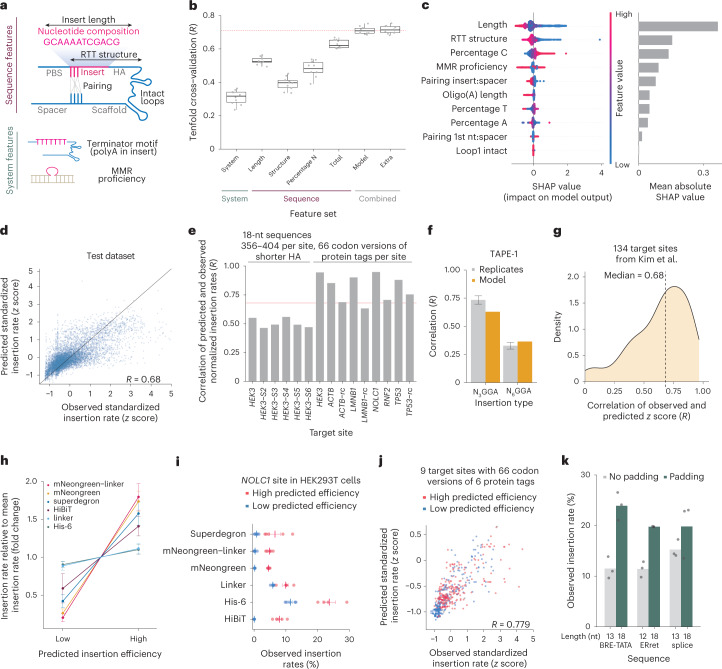
Predicting prime insertion efficiencies. **a**, Schematic representation of model features. **b**, Tenfold cross-validation model performance on the training set (*y* axis) using different feature sets. System: MMR proficiency and Oligo(A) length. Sequence effects: length, reverse transcriptase template (RTT) structure, nucleotide composition and all of them combined (‘Total’). Model: combination of ten features. Extra: 53 features. Dashed line, median of ‘Model’. Box, median and quartiles. Whiskers, 1.5 times interquartile range. **c**, Feature importance. Left, distribution of SHAP values (*x* axis) for each feature (*y* axis, colors). Right, respective mean absolute SHAP values (*x* axis). **d**, Concordance of predicted (*y* axis) and observed (*x* axis) insertion efficiencies on the held-out test set (markers). Solid line, *y* = *x*. Label, Pearson’s *R*. An additional 18 points are beyond the plot limits (Supplementary Fig. 12). **e**, Concordance of predicted and observed values at new sites. Pearson’s *R* between predicted and observed normalized insertion efficiencies (*y* axis) for 356–388 18-nt sequences inserted into six different sites within the HEK3 locus (left bars) and 66 codon variants of six protein tags into nine sites in HEK293T cells (right bars). Line, performance on the dataset from **d**. **f**, Mean replicate correlation (light gray) ±s.e.m. and concordance of predicted and observed rates (yellow) on 6- and 9-nt insertions (63 and 1,908 sequences, respectively) at the TAPE-1 target from (ref. ^42^). **g**, Distribution of Pearson’s *R* between observed and predicted insertion rates (*x* axis) of seven insertions into 134 loci from (ref. ^17^). Dashed line, median. **h**–**j**, Measured insertion rates of predicted high- and low-inserting codon versions of six protein tags into nine sites. **h**, Measurements of insertion rate relative to mean insertion rate of codon sequences (*y* axis, colors) separated into predicted to be highly and lowly inserting (*x* axis). **i**, Insertion rates (*x* axis) of codon variants (markers) of six protein tags (*y* axis) into the NOLC1 site in HEK293T cells. Red, large predicted rate; blue, low predicted rate. Bar and whiskers, mean ± s.e.m. **j**, Concordance of observed and predicted insertion rates of all sequences for all target sites and codon variants. **k**, Effect of padding. Insertion rates (*y* axis) of three sequences (*x* axis) inserted without modification (gray) and padded with optimally predicted sequences to 18 nt (green).

Given our improved understanding of prime insertion rates, we next aimed to predict the relative efficiencies of inserting different sequences into the same site. We extracted 53 salient features such as insert length, nucleotide composition and folding energy for each pegRNA in eight screens (Fig. 5a, Supplementary Table 1 and Supplementary Fig. 11), and used tenfold cross-validation to select an accurate model (Methods and Supplementary Fig. 12). Based on feature correlations, their marginal effect we uncovered above and interpretability, we manually picked a final set of ten features, such that adding the remaining 43 extracted features did not improve the model performance further on the training data (Fig. 5b and Supplementary Fig. 12). The contribution of individual features to prediction reflected the understanding developed above: insert sequence length, the secondary structure of the pegRNA and reverse transcribed sequence, sequence composition and MMR each had a substantial impact, and the direction of these effects was consistent with expectations (Fig. 5c and Supplementary Table 1). The final model trained on the full training set achieved a correlation of 0.68 on held-out sequences, with performance ranging from *R* = 0.44 to 0.92 when restricted to individual screens, exceeding correlation of individual biological replicates in noisier ones (Fig. 5d and Supplementary Fig. 12). We call this method MinsePIE (Modeling insertion efficiency for Prime Insertion Experiments) and incorporated it into a package available at https://github.com/julianeweller/MinsePIE, and produced a web application to predict prime editing insertion rates at https://elixir.ut.ee/minsepie/.

After establishing and interpreting the model, we next tested whether its predictions extrapolate to observations beyond our original screening context (Supplementary Fig. 13). We first measured insertion efficiencies of 356–388 sequences of 18 nt into the *HEK3* and five novel nearby sites, as well as insertions of 66 codon versions of different protein tags in nine novel sites. In spite of new insert sequences, previously unobserved target sites and shorter 15-nt homology arms, the MinsePIE model predicted relative insertion efficacies well, with Pearson’s *R* of 0.46–0.95, compared with replicate reproducibility of *R* = 0.36–0.98 (Fig. 5e and Supplementary Figs. 7b and 14). We then assessed generalizability on external datasets. A recent study by Choi et al. inserted 63 6-nt and 1,908 9-nt sequences (NNNGGA and NNNNNNGGA) into the synthetic, genome-integrated TAPE-1 target sequence using a 13-nt primer binding sequence and a 9-nt homology arm^42^. MinsePIE prediction quality was close to measurement repeatability (*R* = 0.63 and 0.37 for 6-nt and 9-nt insertions, respectively, for prediction versus measurement; *R* = 0.73 and 0.33 for replicate versus replicate; Fig. 5f and Supplementary Fig. 15a,b). Finally, to evaluate MinsePIE performance at many unseen target sites, we predicted the insertion rates of A, C, G, T, AG, AGGAA and AGGAATCATG sequences into 134 loci using pegRNAs with 13-nt primer binding sites and 14-nt homology arms as measured by Kim et al.^17^. The median prediction accuracy for these sites was *R* = 0.68 (range 0.0–0.97; Fig. 5g and Supplementary Fig. 15), which is consistent with the observed model performance on other internal and external datasets.

A predictive model of insertion rate will be useful for experimental optimization, such as selecting the best nucleotide sequence to insert for the common task of tagging endogenous proteins. We used MinsePIE to predict high- and low-performing codon variants of six different protein tags frequently used in molecular biology: His-6, HiBiT^43^, glycine-rich linker, mNeongreen-11 (ref. ^28^), mNeongreen-11 endowed with a linker and a drug-inducible superdegron^44^, to generate in-frame fusions for *ACTB*, *LMNB1*, *NOLC1*, *RNF2* and *TP53* using pegRNAs that targeted both the forward and the reverse strand. We then tested the predicted sequences experimentally and observed a higher relative insertion rate of codon variants predicted to insert well compared with variants predicted to insert at low rates (median fold increase of 1.63; Fig. 5h,i and Supplementary Fig. 14). This demonstrates the advantage of codon-optimization with the MinsePIE model. Beyond grouping into highly and lowly predicted sets, the measured insertion rates of all sequences correlated well with model predictions (*R* = 0.78; Fig. 5j). Finally, since sequences between 15 and 21 nt were inserted more efficiently than 10–14-nt ones, we hypothesized that padding shorter sequences to 18 nt will increase their insertion rates. We used our model to predict optimal padding sequences for three 12–13-nt sequences: a BRE-TATA box element, an endoplasmic reticulum retention (ERret) signal and a consensus splice site, and observed an average increase of 1.4-fold in insertion efficiency when using the padded sequences over the unmodified ones (Fig. 5k). Together, these results demonstrate that our computational model can generalize to novel target sites and can help choose the most efficient sequences to write into the genome.

## Discussion

We presented a comprehensive analysis of prime editing insertion efficiencies using 3,604 pegRNAs and diverse follow-up experiments (summarized in Supplementary Fig. 17). We found that short sequences insert with predictable frequencies across cell lines, target sites, repair contexts and prime editor systems based on their length, cytosine content and tendency to form secondary structure. We discovered that overexpression of the 3′ flap nucleases TREX1 and TREX2 inhibited the insertion of longer sequences, and confirmed that active MMR antagonizes the insertion of shorter ones. The sequence and repair features, through MinsePIE, enable accurate prediction of relative insertion rates for novel sequences, and facilitate optimal design choices for writing short stretches of DNA into genomes.

We uncovered a complex relationship between insertion sequence features and efficiency that is shaped by DNA processing and repair mechanisms. For the shortest sequences of up to 10 nt, it is increasingly appreciated that MMR proficiency is a strong factor^20,35^, and we directly and comprehensively reaffirm this connection here. Surprisingly to us, sequences between 15 and 21 nt could insert at higher rates than shorter ones in MMR-proficient cells, and elongating the insertion can improve its insertion efficacy. This effect is likely due to a combination of antagonization by MMR for the shortest sequences, and the potential steric issues for the 10–14-nt ones.

Sequences longer than 30 nt are incorporated less frequently. This could partly be explained by our discovery that the 3′ flap nucleases TREX1 and TREX2 antagonize prime editing in an insert sequence length-dependent way. One explanation, supported by our observation that more structured long sequences insert at higher frequencies due to factors beyond RNA stability, is that DNA flaps with longer insertions and less structure likely spend more time in a nonhybridized state and expose more single-stranded DNA even when hybridized, thus making them more vulnerable to nuclease degradation. This demonstrates that flap nucleases modulate prime editing, which motivates strategies for the next generation of long sequence insertions.

We further discovered that stronger secondary structure of the pegRNA homology arm and insert sequence led to higher insertion efficiency. This effect was evident when comparing different inserts into the same target, but also explained variable rates when attempting to write the same sequence into different target sites. We observed strong correlations between structure and insertion rates in the context of epegRNAs as well, and correlation was highest when the structure was confined to the insert and the homology arm, indicating that the effects of structures in these two regions are separate. Therefore, we hypothesize that while the epegRNA structure improves editing rates by preventing degradation of the RNA 3′ extension, structure in the transcribed template does so by preventing degradation of the single stranded DNA flap intermediate by flap nucleases. Indeed, flap nucleases had a smaller impact on insertions which resulted in more structured flaps. Alternatively, structured inserts could ease pairing of the edited strand with the nonedited strand due to being sterically smaller via folding onto themselves.

Our improved understanding of insertion efficiency using the prime editing system naturally leads to recommendations for experimental design. First, we suggest choosing sequences with high cytosine content that are prone to form secondary structures. Inserts with runs of adenines should be avoided when using the U6 promoters for pegRNAs. For sequences shorter than 14 nt, transiently inhibiting MMR (as implemented in PE4 or PE5 systems)^20^, or knocking out *MLH1*, will drastically improve insertion rates in MMR-proficient cells. If MMR inhibition is undesired, padding the sequences to 18 nt or installing additional silent mutations on the reverse transcriptase template can increase insertion rates.

We put these recommendations to the test, and greatly improved the efficiency of protein tagging. For example, the His-6 tag, especially if choosing the CAC codon, inserts almost six times as well as the next best tag in our library (Myc-tag). To correct pathogenic deletions, our model can help prioritize targets and pick high-efficiency replacement sequences (for example, through codon variation). We provide empirical measurements on insertion efficiency into multiple target sites for over 100 useful sequences (Supplementary Data 2). For predicting the insertion efficiency of novel sequences, we provide the MinsePIE algorithm as a command-line package^45^ and user-friendly website (https://elixir.ut.ee/minsepie/).

Our study measures thousands of sequences in up to 18 target sites in three cell lines across four prime editor systems. Nevertheless, our insights and the models we built have limitations. First, we measured on-target insertion, and predicted the relative insertion rate of intended sequence, but did not assay genome-wide off-target editing, or model the insertion of nontemplated or mutated sequences that we observed to be rare. Other efforts have comprehensively characterized inserting a small number of edits into a large number of synthetic target sites^17^, and our model performs well to predict the relative efficiency on the majority of these data. A few target sites remained where our model did not perform well and datasets with diverse insertions into many more target sites will be needed to improve the predictions further. While the small number of sites we included limits our ability to model the target site effect, and guide RNA efficacy scores did not account for the target site influence, we believe that some features we uncovered (structure in the reverse transcriptase template, percentage of cytosines, disruption of the scaffold^41^ and so on) also explain differences between efficiencies of pegRNAs more broadly and for edits beyond insertions.

The prime editing field is moving rapidly^15,46^. Diverse applications are already emerging^47^ and some of the most exciting ones are specifically built around the insertion of short sequences. Examples include insertion of recombinase sites using prime editing to enable directed insertion of large DNA cargo of up to 36 kb (refs. ^1,2^), creating long deletions and insertions using paired pegRNAs^1,48–51^, as well as clever utilization of short sequence insertion to generate a molecular recorder for sequential cellular events^42,52,53^. A better understanding of how cellular determinants^20,54^ and pegRNA features affect prime editing rates^17,21^ provides a foundation for these advances. Our work adds the important dimension of short sequence insertion in different DNA repair contexts, which holds promise in enabling both sophisticated genome engineering and the correction of thousands of pathogenic mutations.

## Methods

### Mammalian cell culture

The human HEK293T cell line was purchased from AMS Biotechnology (EP-CL-0005). The HAP1 WT cell line was provided by Andrew Waters (Wellcome Sanger Institute) and the HAP1 *∆MLH1* cell line was purchased from Horizon Discovery (HZGHC000343c022). HEK293T cells were cultured in DMEM (Invitrogen) and HAP1 cells in IMDM (Invitrogen), both supplemented with 10% FCS (Invitrogen), 2 mM glutamine (Invitrogen), 100 U ml^−1^ penicillin and 100 mg ml^−1^ streptomycin (Invitrogen) at 37 °C and 5% CO_2_.

### Primers

All primers used in this study are listed in Supplementary Table 3.

### Plasmid cloning

Plasmids generated in this study are listed in Supplementary Table 4.

*pCMV-PE2-P2A-PuroR* was generated by replacing eGFP from pCMV-PE2-P2A-GFP (Addgene 132776) with PuroR. A gene fragment containing parts of the MMLV reverse transcriptase and the puromycin resistance gene was ordered from IDT (Supplementary Table 5). The gene fragment and pCMV-PE2-P2A-GFP were digested using AgeI, purified with the Monarch PCR & DNA Cleanup Kit (NEB) and ligated with T4 DNA ligase (NEB). The ligation product was transformed into XL10-Gold Ultracompetent Cells (Agilent). Plasmid DNA was isolated using the Plasmid Plus Midi Kit (Qiagen).

*pCMV-PE-FeLV-P2A-EGFP* was generated by replacing the MMLV coding sequence between the XTEN linker and the 2A cleavage peptide with a synthesized gene fragment from IDT using Gibson Assembly which encodes an IDT human codon-optimized version of the MashUp reverse transcriptase (pipettejockey.com) that is engineered from the Feline Leukemia Virus (UniProt Q85521).

*pLentiGuide-BlastR* was generated by replacing the puromycin resistance gene from Lenti_gRNA-Puro (Addgene 84752) with a blasticidin resistance gene. A gene fragment containing parts of the EF1a promoter and the blasticidin resistance gene was ordered from Twist Biosciences (Supplementary Table 5). The gene fragment and Lenti_gRNA-Puro were digested using FseI (NEB) and MluI-HF (NEB), purified with the Monarch PCR & DNA Cleanup Kit (NEB), ligated with T4 DNA ligase (NEB) and transformed into XL10-Gold Ultracompetent Cells (Agilent). Plasmid DNA was isolated using the Qiagen Spin Miniprep Kit.

*pPB-TREG3G-PE2-rtTA3G-P2A-eGFP* was generated by fusing three gene fragments with restriction cloning. The first part contains the ITR sequences for the PiggyBac transposase, the second part contains prime editor 2 under the control of the third-generation doxycycline-inducible rtTA3G promoter and the third part was synthesized by Twist Biosciences and contains a PGK promoter followed by the rtTA3G protein, a P2A sequence and eGFP.

*pTwist_FEN1-T2A-tagBFP*, *TREX1-T2A-mScarlet*, *TREX2-T2A-emiRFP670* and *Acceptor-T2A-eGFP* were ordered from Twist Biosciences in a pTwist EF1 Alpha cloning vector. The protein sequences encoded by the primary transcripts of FEN1, TREX1 and TREX2 were identified on ensembl.org (July 2022), fused with the T2A sequence and the respective fluorophores, and reverse translated into codon-optimized nucleotide sequences (Twist Biosciences).

The pCMV-PE2-P2A-PuroR, pLentiGuide-BlastR and pPB-TREG3G-PE2-rtTA3G-P2A-eGFP plasmids will be made available on Addgene.

### Generating HAP1 cell lines that stably express prime editor

HAP1 cell lines expressing prime editors were generated by cotransfecting pCMV-hyPBase^55^ and pPB-TREG3G-PE2-rtTA3G-P2A-eGFP. First, 500,000 HAP1 WT and 500,000 HAP1 *∆MLH1* cells were each seeded into one well of a six-well plate one d before transfection. For each transfection, 3 µg of each plasmid was mixed with 6 µl of Plus reagent and 7.5 µl of Lipofectamine LTX (Invitrogen) reagent, incubated for 30 min and then added to the cells. At two weeks post transfection, cells were sorted into single clones based on eGFP expression. Two different individual clones were used for each screen.

### Library design

Set 1: The insert sequence libraries contained 2,666 unique sequences, made up of useful molecular biology sequences, the eukaryotic motif library (eukaryotic linear motif, ELM) and sequences with strong secondary structure. We designed four separate versions of this library with identical insert sequences to target the *CLYBL*, *EMX1*, *FANCF* and *HEK3* sites. The pegRNAs contained a 13-nt PBS and a 34-nt homology arm on the reverse transcriptase template. The utility sequences were hand-picked for their usefulness in molecular biology. The ELM instances library with the corresponding fasta file of the genes was downloaded from elm.eu.org/instances.html?q = * (refs. ^26,27^) on 19 November 2020 and filtered to only contain sequences from ‘homo sapiens’ that are longer than one amino acid. The amino acid motifs were extracted from the fasta file based on the indicated start and end sites. Finally, the amino acid motifs were reverse translated into DNA sequence using the ‘reversetranslate’ R package (v.1.0.0) and using the most frequent codon from the ‘homo sapiens’ codon table. For the secondary structure library, 100,000 random DNA sequences of 20- and 30-nt length were generated (RBioinf::randDNA function; v.1.48.0) and their secondary structure was calculated (see the Data analysis and feature generation section). The sequences were distributed into ten bins based on the strength of their secondary structure and 20 sequences were randomly picked from each structure bin to be included in the library. Finally, 30 random perfect 20- and 30-nt RNA hairpins were generated and amended to the secondary structure library. The combined library of insert sequences is included as Supplementary Data 1. The insert sequences were then flanked with primer binding sites, random nucleotide stuffer sequences for shorter inserts, BsmBI sites and target vector compatible overhangs, resulting in 11,166 sequences of 199 nt. The oligonucleotide library was ordered from Twist Biosciences.

Set 2: This set of insert sequences was focused on short sequences between 1 and 10 nt. It included all 1-, 2-, 3- and 4-nt sequences and 100 random sequences (RBioinf::randDNA function; v.1.48.0), respectively, of 5–10 nt, and 61 sequences <10 nt from Set 1 for a total of 999 unique inserts (938 were recovered in screens). The libraries were endowed with target-site-specific adapter sequences and ordered the same way as Set 1.

Eighteen-nt insert sequence libraries: This set of sequences consisted of six sublibraries that were designed to target the *HEK3* site and five additional nearby sites (within 1 kb), dubbed *HEK3-2*, *HEK3-3*, *HEK3-4*, *HEK3-5* and *HEK3-6*. The sublibraries shared 100 identical, randomly generated (RBioinf::randDNA function; v.1.48.0) 18-nt insert sequences and 256–288 sublibrary-specific 18-nt insert sequences that were picked based on their ability to form secondary structure in the reverse transcriptase template. In contrast to Set 1 and Set 2, we ordered oligos for this set of sequences that already included the spacer (20 nt), improved scaffold (86 nt, sequence: gtttaagagctatgctggaaacagcatagcaagtttaaataaggctagtccgttatcaacttgaaaaagtggcaccgagtcggtgc), PBS (13 nt), insert (18 nt) and homology arm (HA) (15 nt). The oligos were endowed with BsmBI sites, overhangs for cloning and primer binding sites for amplification of the oligo pool. The oligonucleotide library was ordered from Twist Biosciences.

Codon variation library: six protein tags, His-6 (HHHHHH), Flag (DYKDDDDK), a glycine-rich linker (GSSGGSSG), the HiBiT tag (VSGWRLFKKIS)^43^, mNeongreen-11 (TELNFKEWQKAFTDMM)^28^ mNeongreen with a linker (GSSGTELNFKEWQKAFTDMM) and a drug-inducible superdegron (LQCEICGFTCRQKGNLLRHIKLH)^44^; were used to tag *ACTB*, *LMNB1*, *NOLC1*, *RNF2* and *TP53* genes, and to insert into the *HEK3* site. We chose *ACTB*, *LMNB1*, *NOLC1* and *RNF2* because they have been successfully edited in the other publications^12^ and *TP53* for its relevance in health and disease. *ACTB*, *LMNB1*, *NOLC1* and *TP53* were tagged at their N termini; an in-frame, internal fusion was made for *RNF2*. For the *ACTB*, *LMNB1* and *TP53* targets, two independent pegRNAs were used that target both the forward and reverse strands (Supplementary Table 6). Because we decided to make in-frame fusions, the position of the insert sequence was shifted up to 6 nt downstream on the reverse transcriptase template relative to the nick. Together, this resulted in nine target sites.

For the His-6 tag and the glycine-rich linker, all possible codon combinations were generated in silico. For the remaining, longer tags, all possible codon variations were generated using only the top two most frequent human codons. MinsePIE was used to predict the insertion efficiencies for the generated codon variants and ten codon variants with both high and low predicted insertion rates were included in the final library. The codon-optimization webtool from Eurofins Genomics (https://eurofinsgenomics.eu/en/gene-synthesis-molecular-biology/geneius/sequence-optimisation/) was used to design an additional version of each tag. This resulted in 594 sequences in total (Supplementary Data 1). The oligos for this set of sequences contained spacer (20 nt), improved scaffold (86 nt, gtttaagagctaagctggaaacagcatagcaagtttaaataaggctagtccgttatcaactcgaaagagtggcaccgagtcggtgc^56^), PBS (13 nt), insert and HA (34 nt). The oligos were endowed with BsmBI sites, overhangs for cloning and primer binding sites for amplification of the oligo pool, and were ordered from Twist Biosciences.

### Library cloning

Set 1 and Set 2: First, a separate, site-specific backbone was cloned for each target site. A gene fragment was ordered containing the protospacer, guide RNA scaffold, parts of the reverse transcriptase template and primer binding site, a stuffer sequence flanked with BsmBI sites for insert library insertion and the T7 terminator motif (Supplementary Table 5). Then, 100 ng of the gene fragments was digested with BsaI-HFv2 (NEB) and purified with the Monarch PCR & DNA Cleanup Kit (NEB). The pLentiGuide-BlastR plasmid was digested with BsmBI-V2 (NEB) at 55 °C for 8 h followed by 20 min of heat inactivation at 80 °C, and gel purified using the QIAEX II Gel Extraction Kit (Qiagen). The gene fragments were ligated into the backbone using T4 DNA ligase (NEB) and transformed into XL10-Gold Ultracompetent bacteria (Agilent). The plasmids were purified with Qiagen Spin Miniprep Kit.

Second, pegRNA insert libraries were inserted into the site-specific backbones. The insert libraries were synthesized as oligonucleotide pools and amplified using KAPA HiFi HotStart ReadyMix (Roche). Libraries for individual target sites were amplified with separate primers (Supplementary Table 3). The products were purified using the Monarch PCR & DNA Cleanup Kit, digested with BsmBI-v2 at 55 °C for 4 h and heat-inactivated at 80 °C for 20 min alongside 5 μg of site-specific plasmids. The digested oligos were purified using the Monarch PCR & DNA Cleanup Kit. The vectors were treated with quick CIP (NEB) for 15 min at 37 °C and then purified using QIAquick PCR Purification Kit (Qiagen). Inserts were ligated into vectors using Golden Gate assembly. A 1:3 molar ratio of insert and vector was mixed with BsmBI-v2 and T4 DNA ligase and incubated in a thermocycler for 30 cycles, alternating between five min at 42 °C and five min at 16 °C and finishing with a heat inactivation step at 60 °C for five min. The ligation products were purified with Monarch PCR & DNA Cleanup Kit and electroporated into MegaX DH10B T1R Electrocomp Cells (Thermo Fisher). The bacteria were grown overnight in liquid culture and plasmid was extracted using the Plasmid Plus Midi Kit. The pegRNA sequences are shown in Supplementary Table 6.

epegRNA libraries were cloned by first generating a *HEK3* site-specific epegRNA backbone with a stuffer sequence for the insert libraries (as above). The tevoprep sequence was added to the fragment containing the protospacer, guide RNA scaffold, parts of the reverse transcriptase template and primer binding site, a stuffer sequence flanked with BsmBI sites for insert library insertion and the T7 terminator motif by PCR (using P42, P43; Supplementary Table 3). Next, the 379 sequences with strong structure were amplified from the Set 1 oligo pool by PCR and cloned into the epegRNA *HEK3* backbone as described above.

Eighteen-nt inserts and codon variation libraries: pLentiGuide-BlastR plasmid was digested with BsmBI-V2 (NEB) at 55 °C for eight h followed by 20 min of heat inactivation at 80 °C and gel purification of the vector using the QIAEX II Gel Extraction Kit (Qiagen). Amplification, purification, digestion and repurification were performed as described above. The oligo sequences were ligated into pLentiGuide-BlastR using Golden Gate assembly, the ligation product was purified and transformed into bacteria, and the plasmid was extracted after an overnight culture as above.

### Lentivirus production

Lentivirus was produced in HEK293FT cells that were transfected with Lipofectamine LTX (Invitrogen). First, 5.4 μg of a lentiviral vector, 5.4 μg of psPax2 (Addgene 12260) and 1.2 μg of pMD2.G (Addgene 12259) were mixed in 3 ml of Opti-MEM together with 12 μl of PLUS reagent and incubated for five min at room temperature. Next, 36 μl of the LTX reagent was added and the mix was incubated for another 30 min at room temperature. Then, 3 ml of the transfection mix was added to 80% confluent cells in 10 ml of DMEM medium in a 10-cm dish. After 48 h the supernatant was collected and stored at 4 °C. Fresh medium was added to the cells and collected 24 h later. The two collections were kept separate. For virus titration, Lenti-X GoStix Plus (Takara) was used following the manufacturer’s protocol.

### pegRNA insertion screens in HEK293T cells

Infection with pegRNA library: Cells were infected with the pegRNA library (separate infections for each target site and library set), aiming at a multiplicity of infection of 0.5 and a guide coverage of >1,000×. Each screen was performed in three biological replicates and independently infected. To achieve this, 6 × 10^6^ cells were plated in three wells of a six-well plate and spin-infected for 15–30 min at 2,000 r.p.m. Following infection, cells were resuspended and replated at 2 × 10^4^ cells per cm^2^. Cells were cultured for seven d and selected for pegRNA integration with 10 µg ml^−1^ blasticidin.

Transfection with prime editors: HEK293T cells were seeded at a concentration of 6.9 × 10^4^ cells per cm^2^ in a 15-cm dish. The next day, the medium was replaced with fresh medium and the cells were transfected using Lipofectamine LTX reagent. Then, 72 µg of PE-Puro or PE-FeLV plasmid was mixed with 8 µg of pCS2-GFP and 40 µl of Lipofectamine P3000 (Invitrogen) in 3.2 ml of Opti-Mem (Gibco). In another tube, 40 µl of Lipofectamine 3000 and 160 µl of Lipofectamine LTX were mixed in 3.2 ml of Opti-Mem. The solutions were combined, incubated for 30 min at room temperature and then added to the cells. For PE3, an additional 6 µg of nicking guide RNA was added. For screens with nuclease overexpression, an additional 30 µg of flap nuclease or eGFP plasmid in the pTwist vectors was added.

### pegRNA insertion screens in HAP1 and HAP1 *∆MLH1* cells

Infection with pegRNA library: The pegRNA library viruses for all target sites and sets were individually quantified using the Lenti-X GoStix Plus (Takara) kit and then combined into one virus pool. The HAP1 and HAP1 *∆MLH1* cells with PiggyBac-integrated PE2 were infected with the virus pool, aiming at a multiplicity of infection of 0.5 and a pegRNA coverage of >1,000×. Each screen was performed in two biological replicates with separate PiggyBac prime editor clones and independently infected. To achieve this, 6 × 10^6^ cells were plated in three wells of a six-well plate and spin-infected for 15–30 min at 2,000 r.p.m. Following infection, cells were resuspended and replated at 2 × 10^4^ cells per cm^2^. Cells were cultured for seven d and selected for pegRNA integration with 10 µg ml^−1^ blasticidin.

For each replicate, 30 million cells were seeded into five-layer flasks and induced with 1 µM doxycycline. The cells were split once at day four and the doxycycline was refreshed. Finally, cells were collected on day seven post induction.

### DNA extraction and library preparation for next-generation sequencing

Genomic DNA extraction and sequencing library preparation for screens were done as described by Allen et al.^10^. Briefly, cell pellets were resuspended in TAIL BUFFER A (100 mM Tris-HCl, 5 mM EDTA, 200 mM NaCl) and then mixed with 1 volume of TAIL BUFFER B (100 mM Tris-HCl, 5 mM EDTA, 200 mM NaCl, 0.4% SDS) supplemented with freshly thawed Proteinase K (20 mg ml^−1^ final). The lysate was incubated overnight at 56 °C. On the next day, RNase A was added to a final concentration of 10 µg ml^−1^ and incubated at 37 °C for 30 min to four h. Then, 1 volume of isopropanol was added and the DNA spooled on a sterile inoculation loop. The DNA was washed three times by dipping it into consecutive 5-ml tubes containing 70% ethanol. The DNA was air-dried for 5–10 min and resuspended in TE buffer (pH 8.0).

For each screen, two independent amplicons were generated by PCR using Q5 HotStart High-Fidelity 2X Master Mix (NEB). One amplicon was for the targeted locus and one amplicon for the pegRNA locus (primers in Supplementary Table 3). To maintain high coverage for each sample, 40 μg of genomic DNA was used as the template and each PCR reaction was run in 50-μl aliquots containing no more than 5 μg of genomic DNA. The PCR reactions were column-purified using the QIAquick PCR Purification Kit (Qiagen). Sequencing adapters and barcodes were added with a second round of PCR using the KAPA HiFi HotStart ReadyMix (Roche), primers P3 and P4 (Supplementary Table 3) and 1 ng of template DNA. Amplicons were purified with Agencourt AMPure XP beads in a 0.7:1 ratio (beads to PCR reaction volume) and quantified with the Quant-iT High-Sensitivity dsDNA Assay Kit (Invitrogen). The amplicons were pooled together and sequenced on the Illumina HiSeq 2500 using HiSeq Rapid SBS Kit v2 (500 cycles, 250 paired-end).

### Reverse transcription of pegRNA libraries

Frozen cell pellets containing 4.5–6.1 million cells from screens targeting the *HEK3* site in HEK293T cells were washed with 500 µl of PBS and the RNA was extracted using the mirVana miRNA Isolation Kit (Invitrogen). Then, 8.4–16.6 µg of template RNA split across eight reactions was used for genomic DNA digestion and complementary DNA synthesis with the SuperScript IV VILO Master Mix with ezDNase (Invitrogen). For cDNA synthesis, a primer was used that was reverse complementary to the 13-nt PBS with extra nucleotides on the 5′ end (italic) to provide additional base pairing for PCR amplification (*ATCGAGTTT*CAGACTGAGCACG; Supplementary Table 3). pegRNAs were amplified from the cDNA mixture by 27 cycles of PCR using KAPA HiFi HotStart ReadyMix (Roche) and primers P39 and P40 (Supplementary Table 3). Library preparation and sequencing were performed as described in the DNA extraction and library preparation for next-generation sequencing section.

### Generating read count tables

Paired forward and reverse reads from Illumina sequencing were merged using PEAR v.0.9.11. Data for the same screen but different sequencing lanes were concatenated. The resulting merged fastq files were processed using a custom R script (read_match_pegRNAs.R, GitHub^45^). First, DNA sequences were trimmed to contain the 10 nt up- and downstream of the nick site (for target site amplicon) or to contain 15 nt up- and downstream of the nick site (pegRNA amplicon). On average, 98% of reads were matched for the target site amplicon and 84% for the pegRNA amplicon. The trimmed sequences were then matched to each insert in the pegRNA library flanked by 10 nt of target site sequence (for target site amplicon) or flanked by 15 nt of pegRNA plasmid sequence (pegRNA amplicon), requiring 0 mismatches. Adding the flanking sequences ensures that only insertions at the correct locations are considered. On average, 92% of reads were matched to the unedited locus or an insertion for both the target site amplicon and the pegRNA amplicon.

### Combining replicates

pegRNAs where any replicate had fewer than 20 reads in the pegRNA amplicon mapping to it were filtered out. Insert counts were normalized to frequencies by dividing the reads for each insert by the number of reads in each screen. Insertion efficiencies were calculated for each replicate and screen by dividing the target insert frequency by the pegRNA insert frequency. (Note: calculating insertion frequencies this way likely underestimates them, as it does not take cells that were not infected with the library into account. In addition, an average of 16% of reads in the pegRNA amplicons did not match to any sequence in the library.) Finally, insertion efficiencies were averaged across replicates. The script used to combine replicates is available on GitHub^45^ as ‘combine_replicates.R’. The processed read count tables are shown in Supplementary Data 2.

### Mutation rates around the insertion site and indel detection

The fastq reads of the target sites were trimmed by matching a stretch of 10 nt directly upstream of the PBS and 60 nt downstream of the insertion site (*CLYBL*: CTGAATGGTG, CAGAGTTCCA; *EMX1*: GGGCCTGAGT, ATGGGGAGGA; *FANCF*: CCTCATGGAA, AGCACCTGGG; *HEK3*: CCTTGGGGCC, AGCTTTTCCT). The occurrence of library insertions was detected by pattern matching the trimmed reads for library sequences. Indel detection: The trimmed reads were filtered in a series of steps. First, sequences with insertions at the nick site that perfectly match a sequence in the insert libraries were removed (this also means that our method cannot detect single/double/triple-nucleotide insertions at the nick site because our library contains all possible singlets/doublets/triplets). Second, sequences that contained ‘N’ were removed. Third, sequences with a perfectly preserved sequence around the cut site were removed. Fourth, sequences that were 83-nt long were removed (83 nt corresponds to the length of a sequence without indels). The remaining sequences were annotated according to the indel type. Scaffold integrations were sequences that contained five or more nucleotides of the scaffold (GCACC) directly downstream of the reverse transcriptase template. Mutated insertions were sequences that matched any sequence >10 nt in the library with no more than three mismatches (fuzzyjoin R package v.0.1.6, optimal string alignment method). Duplications were sequences that contained two or more copies of the homology arm sequence. Deletions at the target sites were deletions that overlapped up to 10 nt up- and/or downstream with the nick site. Other deletions were deletions that did not overlap with the nick site and all remaining sequences are classified as ‘other’. The scripts used to call mutation rates and indels are available on GitHub^45^ as ‘find_mutations.R’.

SNV detection: Going from the outside to the inside of the trimmed sequence (with the nicking site being between the two innermost nucleotides), the occurrence of the four nucleotides was counted at every position. Nonreference nucleotides were classified as mutations with the exception of a nonreference SNP (A) in HEK293T cells for one of three alleles at position +9. The reverse transcriptase template on the pegRNA corresponds to the sequence of the major allele (G).

### Data analysis and feature generation

Merging data from Set 1 and Set 2: For each target site and cell line, the insertion rates in Set 2 were multiplied by the ratio of the mean insertion rate of the shared sequences in Set 1 and the mean insertion rate in Set 2. For the 140 shared insert sequences, the mean insertion rate between both sets was calculated. Length-normalized insertion rates: Length residuals were calculated by dividing the insertion rate by the median insertion rate for sequences of the same length (for sequences <10 nt) or by dividing sequences into length bins. The length bins consisted of sequences of 10–14, 15–19, 20–24, 25–29, 30–39, 40–49, 50–59 and 60–69 (sequences with lengths above 30 nt were divided into length bins of 10 nt because there were fewer longer sequences in the library). The melting temperature for the insert sequence was calculated using SeqUtils.MeltingTemp.Tm_NN from biopython. The RNA fold (v.2.4.16) algorithm of the ViennaRNA (v.2.5.0a) package^57,58^ was used to calculate the tendency of insert sequences (alone or in the context of PBS and/or HA) to form secondary structures. The free energy was normalized to the mean and standard deviation (*z* score) of 1,000 random sequences with the same length and in the same context.

The 6-nt and 9-nt insertion data from Choi et al.^42^ were filtered for sequences with more than 20 sequencing reads for each pegRNA replicate and more than 30 sequencing reads for the plasmid reads, followed by feature calculation as described above. The insertion and plasmid read frequencies were calculated as the fraction of insertion mapping reads in all reads, and the normalized insertion rate as the ratio of insertion read frequency to the plasmid read frequency normalized to the mean and standard deviation of each dataset (*z* score). The data from Kim et al. were filtered to contain target sites with all seven insertions and no other edits, followed by feature calculation as described above. Edit rates were normalized to the mean and standard deviation of editing rates at each target site.

### Comparison of HAP1 and HAP1 *MLH1* lines

To account for screen batch effects for direct comparisons (Fig. 2f and Supplementary Fig. 2d), the mean insertion rates across wild-type and *MLH1* knockout HAP1 cell lines were scaled to be identical for >13-nt sequences that are not affected by MMR. The fold changes of the scaled insertion efficiencies between HAP1 *∆MLH1* and HAP1 lines were then calculated for each sequence in the library.

### Validation of nuclease overexpression with individual pegRNAs

We chose four different insertions (C, CAG, a BCL6 recognition sequence: TTCTAGGAA and a Myc-tag: GAGCAGAAGCTGATCAGCGAAGAGGACCTC) from our pooled library for validation and cloned them into *HEK3* site-targeting pegRNAs endowed with 25- or 34-nt homology arms. At one d before transfection, HEK293T cells were seeded in two 24-well plates at 50,000 cells per well. All transfections were done in replicates and each well was transfected with 500 ng of pCMV_PE2_P2A_PuroR, 150 ng of pTwist nuclease or eGFP overexpression constructs, and 100 ng of pegRNA using Lipofectamine LTX according to the manufacturer’s protocol. Successful transfection one d later was confirmed by fluorescence microscopy and 2 µg ml^−1^ puromycin was added one d later. Cells were collected five d post transfection by direct lysis of cell pellets using home-made quick extract buffer (1 mM CaCl_2_, 3 mM MgCl_2_, 1 mM EDTA, 1% Triton X-100, 10 mM Tris pH 7.5) with freshly added proteinase K (0.2 mg ml^−1^) followed by 15 min of incubation at 65 °C and 20 min of incubation at 95 °C. Then, 1.5 µl of the lysate was directly added to 25 µl of amplicon PCRs. Sequencing adapters and barcodes were added by a second round of PCR and the purified products were sequenced on an Illumina Miseq (300 cycles). Correctly edited reads were identified by pattern matching for the insert sequence flanked by 10 nt of the target site to each end. Unedited sequences were detected by matching the 20 nt of wild-type sequence around the nick site. The insertion rate was calculated by dividing the number of edited reads by the number of wild-type reads.

### Modeling

Insertion efficiencies were normalized (*z* score) between screens and replicates by subtracting the corresponding mean insertion efficiency from each individual insertion efficiency and dividing it by the standard deviation of the insertion efficiency. Categorical features were one-hot encoded. Hyperparameters were tuned for each model by evaluating average model performance after fivefold cross-validation using each combination of hyperparameters, then choosing the parameter combination resulting in the best cross-validation performance. The Lasso and Ridge regressions were tested with alpha values of 0, 0.00001, 0.0001, 0.001, 0.01, 0.05, 0.1, 0.2, 0.3, 0.4, 0.5, 0.6, 0.7, 0.8, 0.9 and 1. The Random Forest regressor was tested with n_estimators of 5, 10, 50, 100, 500 and 1000; max depth of 2, 5, 7, 10 and None; and min_samples_leaf of 1, 5 and 10. The Multilayer perceptron regressor was tested with hidden_layer_size of (10), (100), (100, 10), (1000, 100) and (1000, 100, 10); and alpha of 0.01, 0.1, 0.5 and 1. The gradient boosted tree from XGBoost^36^ was tested with n_trees of 1, 5, 10, 50, 100, 500 and 1000; max_depth of 1, 2, 3, 4, 5, 7 and 10; l1_penalty and l_2 penalty of 0, 0.001, 0.01, 0.1, 0.5 and 1; colsample of 0.1, 0.3, 0.5, 0.7, 0.9 and 1; gamma of 0 .001, 0.01, 0.1, 0.5 and 1; and learning_rate of 0.0001, 0.001, 0.01, 0.1, 0.3 and 0.5. The scikit-learn models were trained using parameters obtained from hyperparameter tuning: Lasso regression was performed with alpha = 0.1; Ridge regression was performed with alpha of 0.01; Random forest had no maximum depth, 1000 estimators and min_samples_leaf of 5; Multilayer perceptron regressor was trained with alpha = 1, 200 maximum iterations at a constant learning rate of 0.001, a hidden layer size of (1000, 100) and ‘lbfgs’ solver. Gradient boosted tree from XGBoost^59^ was trained with a minimum loss reduction of 0.1, 100 trees, a learning rate of 0.1, maximum depth of 4, 0.00001 L1 regularization on weights, 0.1 L2 regularization on weights and a subsample ratio of one per column when constructing each tree.

The final model was trained with XGBoost using the features length; normalized secondary structure of the reverse transcriptase template; MMR proficiency; percentage of the nucleotides C, A and T; the number of paired bases between the first 3 nt of the insert and the last 3 nt of the spacer in addition to the first nucleotide of the scaffold; complementarity between the first nucleotide of the insert and the nucleotide at the nicking site; the maximum number of consecutive adenines in the insert; and the intactness of loop1. Features in each set are summarized in Supplementary Tables 1 and 2.

For training, unique insert sequences were split randomly into training and test sequences at a ratio of 0.7 (Supplementary Fig. 10a). Measurements for different target sites and cell lines were assigned to training and test data based on the grouping of insert sequences. The model was trained and predictions were evaluated using Pearson’s *R* based on the correlation between test data and corresponding predictions. SHapley Additive exPlanations (SHAP) values for the model and feature importance for the prediction of specific outcomes were calculated using the SHAP TreeExplainer and explainerModel^60^.

### Statistics and reproducibility

The *n* numbers denoted in the figure legends refer to independent experiments that were separately infected with the pegRNA library. Measurements were always taken from distinct samples. No statistical methods were used to predetermine sample size. The experiments were not randomized and the investigators were not blinded to allocation during experiments and outcome assessment. Wherever correlations were indicated, Pearson’s *R* was used. The *t*-tests (Supplementary Fig. 5a,b) were performed as two-sided tests. Normal distribution of the underlying data was assumed and no adjustments for multiple comparisons were made.

### MinsePIE website

The MinsePIE website uses the MinsePIE package available at https://github.com/julianeweller/MinsePIE to serve as a user-friendly and interactive way to predict insertion efficiency (Supplementary Fig. 10b). There are three main modes, with standard highlighting all relevant sequence features, manual allowing more advanced usage where the user can adjust relevant parameters (for example, mean and s.d. of editing rate) and batch mode allowing to upload a set of sequences for analysis. A table highlighting insert sequences, respective *z* scores and insertion prediction scores is given in each usage mode. For ease of analysis, color codes are used in the table and the following distribution graph to highlight the sequences with the highest insertion efficiency scores. MinsePIE web application makes use of Vue.js (v.2.6.11), D3.js (v.3.5.17) and agGrid (v.24.1.1) libraries and the Flask framework (v.2.0.2). Genomic data are retrieved via https://api.genome.ucsc.edu.

### Padding of shorter insert sequences

Three sequences between 12 and 13 nt (an endoplasmic reticulum retention signal, AAGGACGAGCTG; a BRE-TATA element, CCACGCCTATAAA; and a consensus splice motif, TTTTTTTCAGGTT) were chosen for padding. The sequences were padded to 18 nt with all possible nucleotide combinations. MinsePIE was used to predict the insertion rates for these variants at the *HEK3* site. The sequences with highest predicted efficiencies were picked for testing: CAAGGACGAGCTGTCCAC, CCCACGCCTATAAAGGCC and GCTTTTTTTCAGGTTCTC. The padded and original inserts were endowed with a 13-nt PBS and 34-nt reverse transcriptase template and cloned into the pU6-pegRNA-GG-acceptor (Addgene no. 132777) as described previously^12^. Editing efficiencies were assessed by transient transfection in an arrayed format. Therefore, 10,000 HEK293T cells were seeded into a 96-well plate in triplicates. On the following day, 50 ng of pegRNA plasmids and 200 ng of pCMV-PE2-PuroR were transfected using 0.3 µl of Lipofectamine LTX (Thermo Fisher Scientific) and 0.1 µl of Plus reagent per well according to the manufacturer’s instructions. After one d, 2 µg ml^−1^ Puromycin was added. Cells were collected four d post transfection by direct lysis of cell pellets using home-made quick extract buffer (1 mM CaCl_2_, 3 mM MgCl_2_, 1 mM EDTA, 1% Triton X-100, 10 mM Tris pH 7.5) with freshly added proteinase K (0.2 mg ml^−1^) followed by 10 min of incubation at 65 °C and 15 min of incubation at 95 °C. Then, 3 µl of the lysate was directly added to amplicon PCRs. Sequencing adapters and barcodes were added by a second round of PCR and the purified products were sequenced on an Illumina Miseq (300 cycles). Correctly edited reads were identified by pattern matching for the insert sequence flanked by 10 nt of the target site to each end. Unedited sequences were detected by matching the 20 nt of wild-type sequence around the nick site. The insertion rate was calculated by dividing the number of edited reads by the number of wild-type reads.

### Software

The software used comprised BaseSpaceCLI (v.1.4.0); Geneius codon-optimization webtool from Eurofins Genomics (accessed 2022); PEAR (v.0.9.11); Python (v.3.8.10); Python packages: Biopython (v.1.79), more-itertools (v.8.5.0), pandarallel (v.1.6.1), scikit-learn (v.0.24.2), scipy (v.1.5.3), shap (v.0.39.0), statannot (v.0.2.3) and XGBoost (v.1.4.0); R (v.4.0.2); ViennaRNA (v.2.5.0); and R packages: Broom (v.0.7.9), fuzzyjoin (v.0.1.6), ggpointdensity (v.0.1.0), RBioinf (v.1.48.0), reversetranslate (v.1.0.0), ShortRead (v.1.46.0), spgs (v.1.0–3), Tidyverse (v.1.3.1) and Viridis (v.0.6.1).

### Reporting summary

Further information on research design is available in the Nature Portfolio Reporting Summary linked to this article.

## Online content

Any methods, additional references, Nature Portfolio reporting summaries, source data, extended data, supplementary information, acknowledgements, peer review information; details of author contributions and competing interests; and statements of data and code availability are available at 10.1038/s41587-023-01678-y.

### Supplementary information

Supplementary Information Supplementary Figs. 1–18, Note and Tables 1–4.

Reporting Summary

Supplementary Tables 5 and 6 Supplementary Table 5: Gene fragments. Supplementary Table 6: pegRNA used in this study.

Supplementary Data 1 All insert sequences tested in the libraries.

Supplementary Data 2 Editing results for different insertion sequences in the pooled prime insertion screens.

## Data Availability

Read count tables for all screens, mutation frequencies at each position and sequences with indels are shown as Supplementary Data files. Figures with associated raw data: Figs. 1–5 are associated with Data_2_insertion_frequencies.

## Code availability

Scripts and models are made available on https://github.com/julianeweller/MinsePIE (ref. ^61^). The MinsePIE website is available at https://elixir.ut.ee/minsepie.
