# Supplementary material for: Prediction of prime editing insertion efficiencies using sequence features and DNA repair determinants
Source: Nat Biotechnol. 2023 Feb 16;41(10):1446–56. doi: 10.1038/s41587-023-01678-y (PMC10567557; doi:10.1038/s41587-023-01678-y)
Supplement: Supplementary file 1 — Supplementary Figs. 1–18, Note and Tables 1–4. [file 41587_2023_1678_MOESM1_ESM.pdf]

nature biotechnology

Article

https://doi.org/10.1038/s41587-023-01678-y

Prediction of prime editing insertion efficiencies using sequence features and DNA repair determinants

In the format provided by the authors and unedited

Supplementary Information

Supplementary Figures:

Supplementary Figure 1. Reproducibility of insertion efficiencies.
Supplementary Figure 2. Characterization of unintended editing outcomes.
Supplementary Figure 3. Prime insertion screen in MLH1 knockout HAP1 cells.
Supplementary Figure 4. The determinants of pegRNA expression levels and their influence on insertion rate.
Supplementary Figure 5. Alternative prime editor systems have consistent insertion patterns.
Supplementary Figure 6. Flap nuclease overexpression affects insertion rates.
Supplementary Figure 7. Nucleotide composition affects insertion efficiencies.
Supplementary Figure 8. pegRNA structure affects insertion efficiencies.
Supplementary Figure 9. Longer sequences benefit more from the additional structure.
Supplementary Figure 10. Structure and cytosine content explain why some sequences are inserted better than others.
Supplementary Figure 11. Data splitting into test and train sets.
Supplementary Figure 12. Model architectures and features.
Supplementary Figure 13. Editing contexts for all datasets.
Supplementary Figure 14. Replicate correlation for insertions of protein tags into new targets.
Supplementary Figure 15. Predicting high- and low-inserting codon versions of protein tags.
Supplementary Figure 16. Predicting high- and low-inserting codon versions of protein tags.
Supplementary Figure 17. Overview of the pathway, features, libraries, and screens explored in this study.
Supplementary Figure 18. Sequencing coverage for all screens.

Supplementary Note 1: Codon choice for insertion sequences

Supplementary Tables:

Supplementary Table 1. Explanation of features included in the MinsePIE model
Supplementary Table 2. Feature sets
Supplementary Table 3. Sequences of oligonucleotides used in this study
Supplementary Table 4. Plasmids used in this study
Supplementary Table 5. Gene fragments
Supplementary Table 6. pegRNAs used in this study

Supplementary Data Files:

Supplementary Data 1. Insert sequence library
Supplementary Data 2. Insert sequence frequencies (read count table)

Supplementary Code:

Scripts used to analyze screen data: https://github.com/julianeweller/MinsePIE

Supplementary Figures

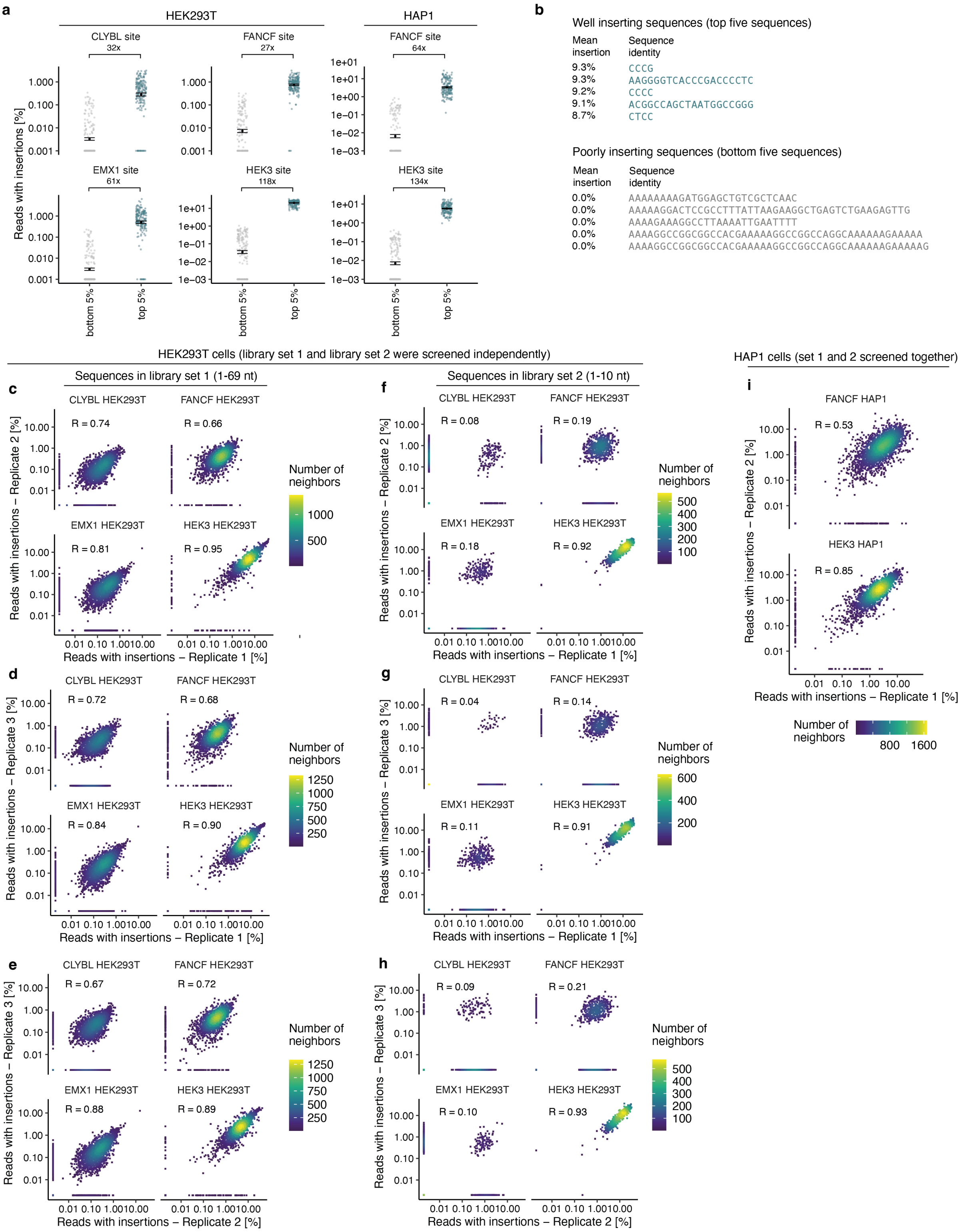

Supplementary Figure 1. Reproducibility of insertion efficiencies. a. Screen normalized insertion efficiency (y-axis) for the top 5% of pegRNAs with the highest insertion rates across all screens and the bottom 5% of pegRNAs (x-axis, colors). Markers are individual pegRNAs. Data

are presented as mean values +/- SEM. n=3 biological replicates. **b.** Example of well and poorly inserting sequences with their respective mean insertion rates across screens. **c.** Percent insertion in replicate 1 (x-axis) compared to percent insertion in replicate 2 for insert sequences of the library Set1 (markers) for different target sites (panels) in the HEK293T cell line. **d-e.** As in (c) but for different replicate comparisons. **f-h.** As in (c-e) but for the library Set2. **i.** As in (c) but for the HAP1 cell line and screening library set 1 and library set 2 together.

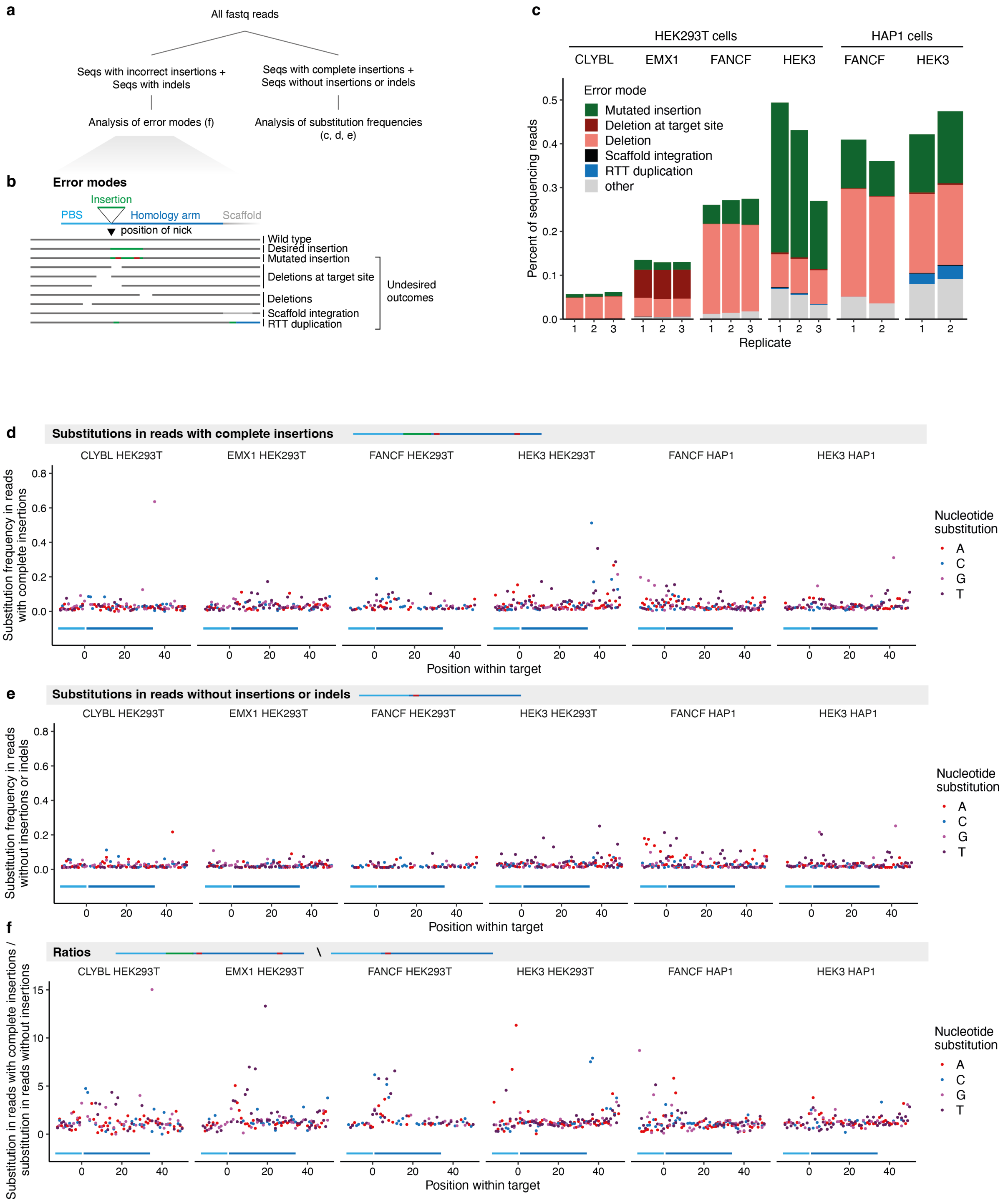

Supplementary Figure 2. Characterization of unintended editing outcomes. a. Schematic of the analysis for unintended outcomes. **b.** Schematic of the various analyzed error modes. RTT: reverse transcriptase template. **c.** Frequencies of unintended outcomes (y-axis) stratified by error types (colors) for replicates (x-axis) at various target sites and cell lines (panels). **d.** The average percentage of sequencing reads with complete library insertions (y-axis) with a non-reference sequence nucleotide (colors) at positions relative to the nicking site (x-axis). *n*=3 biological replicates for HEK293T cells and *n*=2 biological replicates for HAP1 cells. **e.** As (d) but instead showing reads without insertions or indels. **f.** As (d) but displaying the fold-changes between the averages for reads with complete insertions and for reads without insertions or indels.

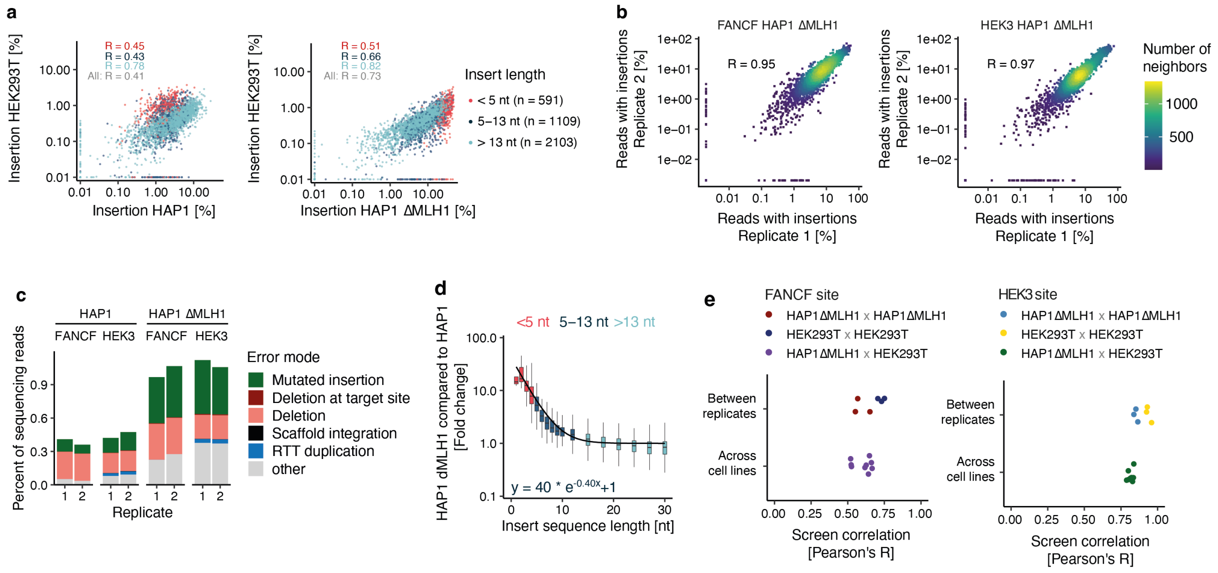

Supplementary Figure 3. Prime insertion screen in MLH1 knockout HAP1 cells. a. Insertion rate in one cell context (y-axis) compared to in another context (x-axis) at the FANCF target of individual sequences (markers), comparing HEK293T to HAP1 cells (left panel) and HEK293T cells to HAP1 ΔMLH1 cells (middle panel). Red: short sequences (up to 4 nt); blue: medium sequences (5-13 nt); teal: longer sequences (>13 nt). Label: R between rates. The data are an average from *n*=3 biological replicates (HEK293T) or *n*=2 biological replicates (HAP1 and HAP1ΔMLH1). **b.** Replicate correlation for HEK3 (upper panel) and FANCF (lower panel) target sites in the HAP1 ΔMLH1 cell line. Displaying percent insertion in replicate 1 (x-axis) compared to percent insertion in replicate 2 (y-axis) for insert sequences (markers). **c.** Frequencies of unintended outcomes (y-axis) stratified by error types (colors) for replicates (x-axis) at various target sites and cell lines (panels). **d.** The ratio of relative insertion rates (Methods) at the FANCF locus between HAP1 ΔMLH1 and HAP1 cells (y-axis) for different lengths (x-axis) stratified by colors as in a). Box plot: median and quartiles; whiskers: least extreme of 1.5 times the interquartile range from the quartile value and the most extreme value. Line: fit from an exponential model (ratio ~ a * exp(-b*length)+1, black line). *n*=2 biological replicates. **e.** Replicate concordance and concordance between different cell lines. Pearson's R between insertion rates in two screens (x-axis) for different comparisons (y-axis, colors). Markers: correlation value of one pair of screens.

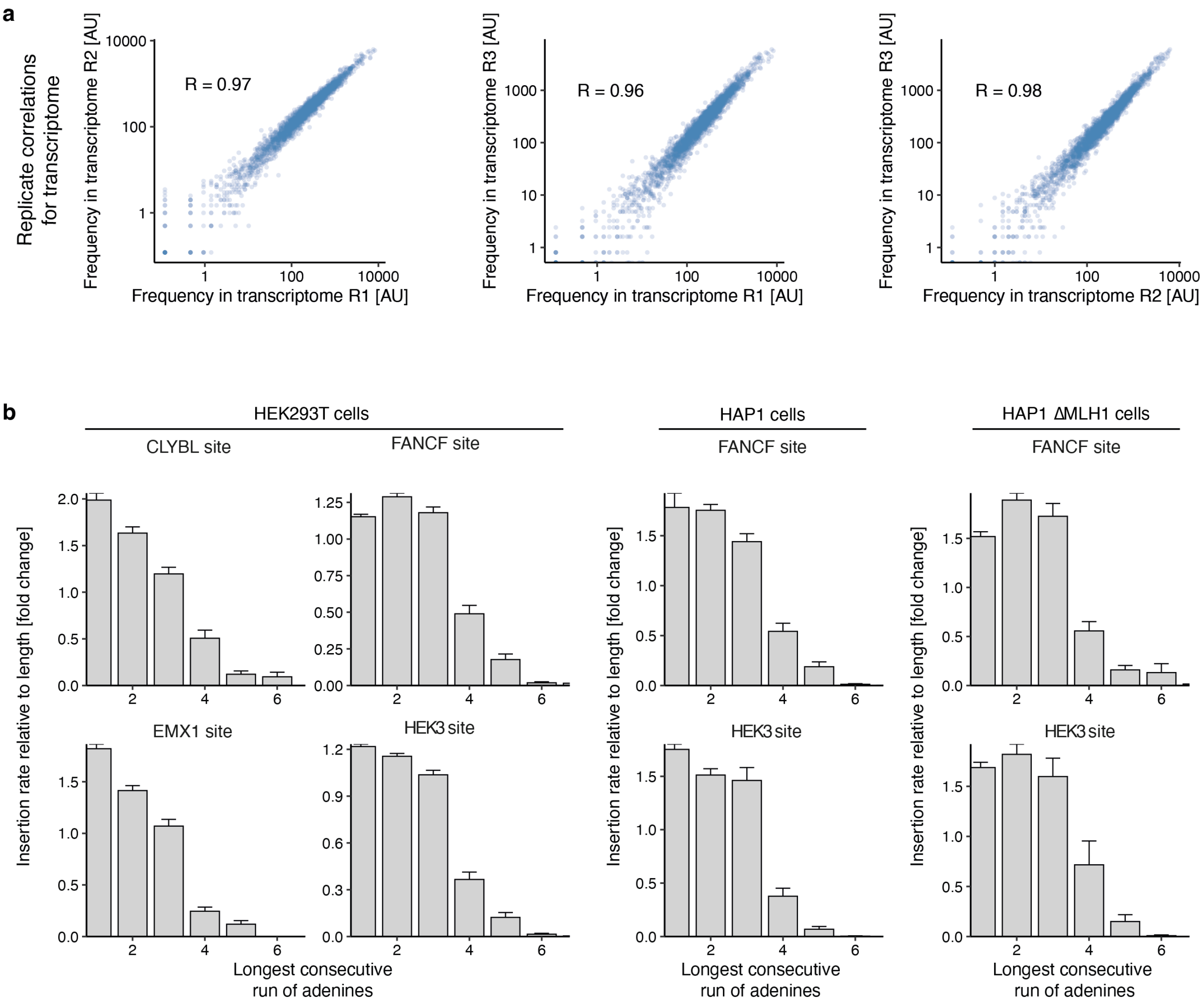

Supplementary Figure 4. The determinants of pegRNA expression levels and their influence on insertion rate. a. Normalized pegRNA read counts from the transcriptome in one replicate (x-axis) compared to another replicate for insert sequences (markers) for different pairwise combinations (panels). **b.** Average insertion rate relative to length bin median (y-axis) for inserts stratified by the longest consecutive run of adenines (x-axis). Panels show various target sites and cell lines. Data are presented as mean values +/- SEM. *n*=3 biological replicates for HEK293T cells and *n*=2 biological replicates for HAP1 and HAP1*ΔMLH1* cells.

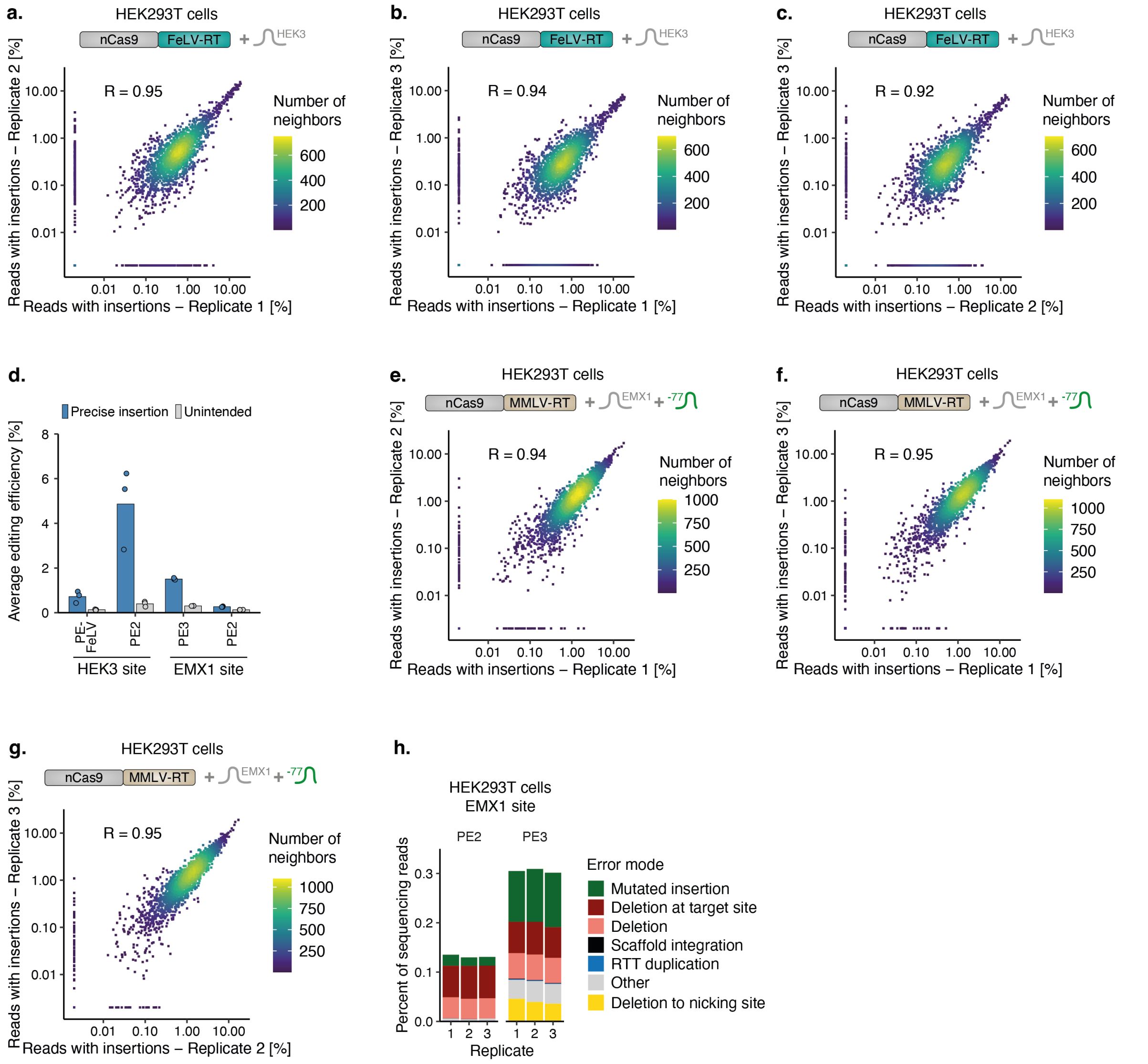

Supplementary Figure 5. Alternative prime editor systems have consistent insertion patterns. a. HEK293T cells expressing nicking Cas9 fused to the Feline Leukaemia Virus Reverse Transcriptase. Comparing percent reads with insertions in replicate 1 (x-axis) to replicate 2 (y-axis) for library Set1 insert sequences targeting the HEK3 site (markers). **b.** As (a) but comparing replicates 1 and 3. **c.** As (a) but comparing replicates 2 and 3. **d.** Editing frequencies for alternative prime editing systems. Mutation frequency (y-axis) for three biological replicate screens (markers) using different prime editor systems (x-axis) stratified by mutation type (blue: insertions; gray: unintended outcomes). Bar: average of markers. **e.** HEK293T cells expressing PE2 and a nicking guide RNA that targets 77 nt downstream. Comparing percent reads with insertions in replicate 1 (x-axis) to replicate 2 (y-axis) for library Set1 insert sequences targeting the EMX1 site. **f.** As (e) but comparing replicates 1 and 3. **g.** As (e) but comparing replicates 2 and 3. **h.** Frequencies of unintended outcomes (y-axis) stratified by error types (colors) for replicates (x-axis) at the EMX1 target sites comparing cells without nicking guide RNA (PE2) and with nicking guide RNA (PE3) (panels).

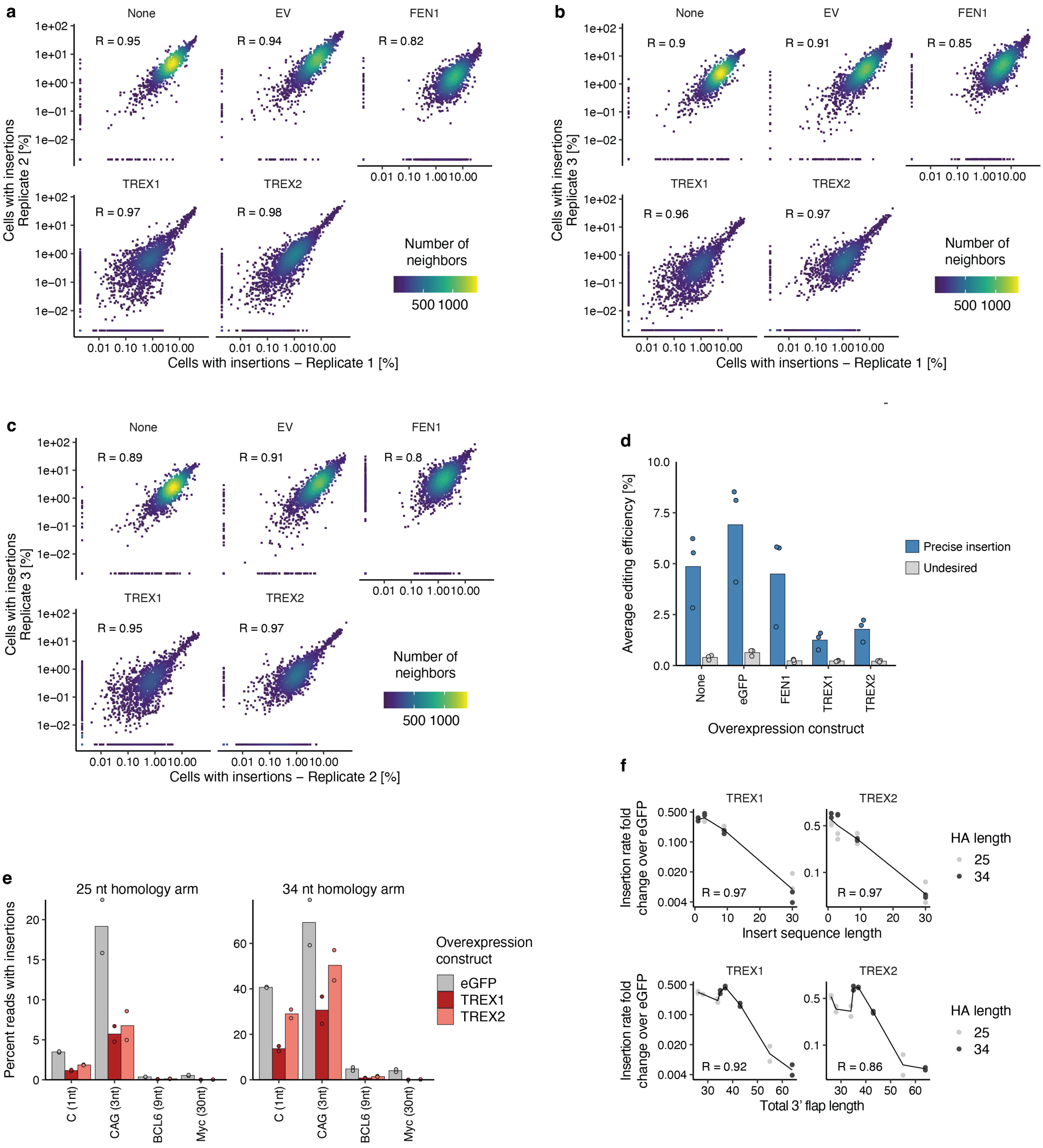

Supplementary Figure 6. Flap nuclease overexpression affects insertion rates. a. HEK293T cells overexpressing various constructs (panels). Comparing percent reads with insertions in replicate 1 (x-axis) to replicate 2 (y-axis) for library Set1 insert sequences targeting the HEK3 site. **b.** As (a) but comparing replicates 1 and 3. **c.** As (a) but comparing replicates 2 and 3. **d.** Editing frequencies for screens with overexpression constructs. Mutation frequency (y-axis) for three biological replicate screens (markers) using different prime editor systems (x-axis) stratified by mutation type (blue: insertions; gray: unintended outcomes). Bar: average of markers. **e.** Insertion frequencies (y-axis) of four sequences with varying insert lengths (x-axis) for two biological replicates (markers) while overexpressing eGFP, TREX1, or TREX2 (colors), stratified by homology arm lengths (panels). Bar: average of markers. **f.** Insertion rate fold changes (y-axis) over eGFP in cells overexpressing TREX1 or TREX2 (columns) for sequences stratified by insert sequence length (x-axis, top row) or by the length of the total 3'flap (x-axis, bottom row) from two biological replicates (markers). Bar: average of markers.

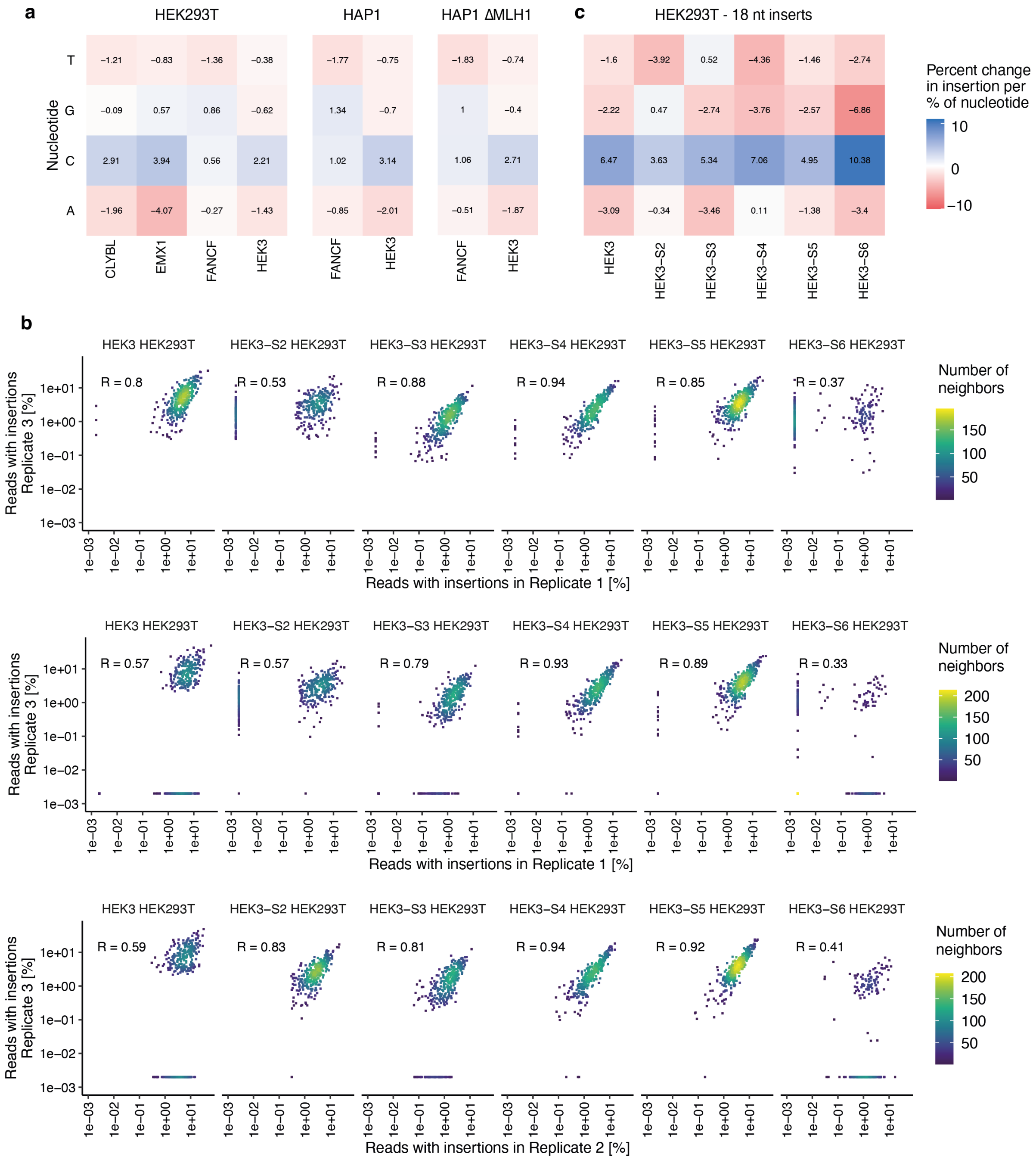

Supplementary Figure 7. Nucleotide composition affects insertion efficiencies. a. Percent additional insertion relative to the average length-normalized insertion rate (color) per extra percent of nucleotide in the insert sequence (y-axis) across different cell lines and targets (x-axis). Data represent the average of n=3 biological replicates. **b.** Percent insertion in one replicate (x-axis) compared to percent insertion in another replicate for the new set of screens with 18 nt insert sequences (markers) at different target sites (rows) within 1 kb of the HEK3 site in HEK293T cells. **c.** As (a) but for but for a new set of screens with 18 nt inserts and 15 nt homology arms targeting five novel sites within 1 kb of the HEK3 site.

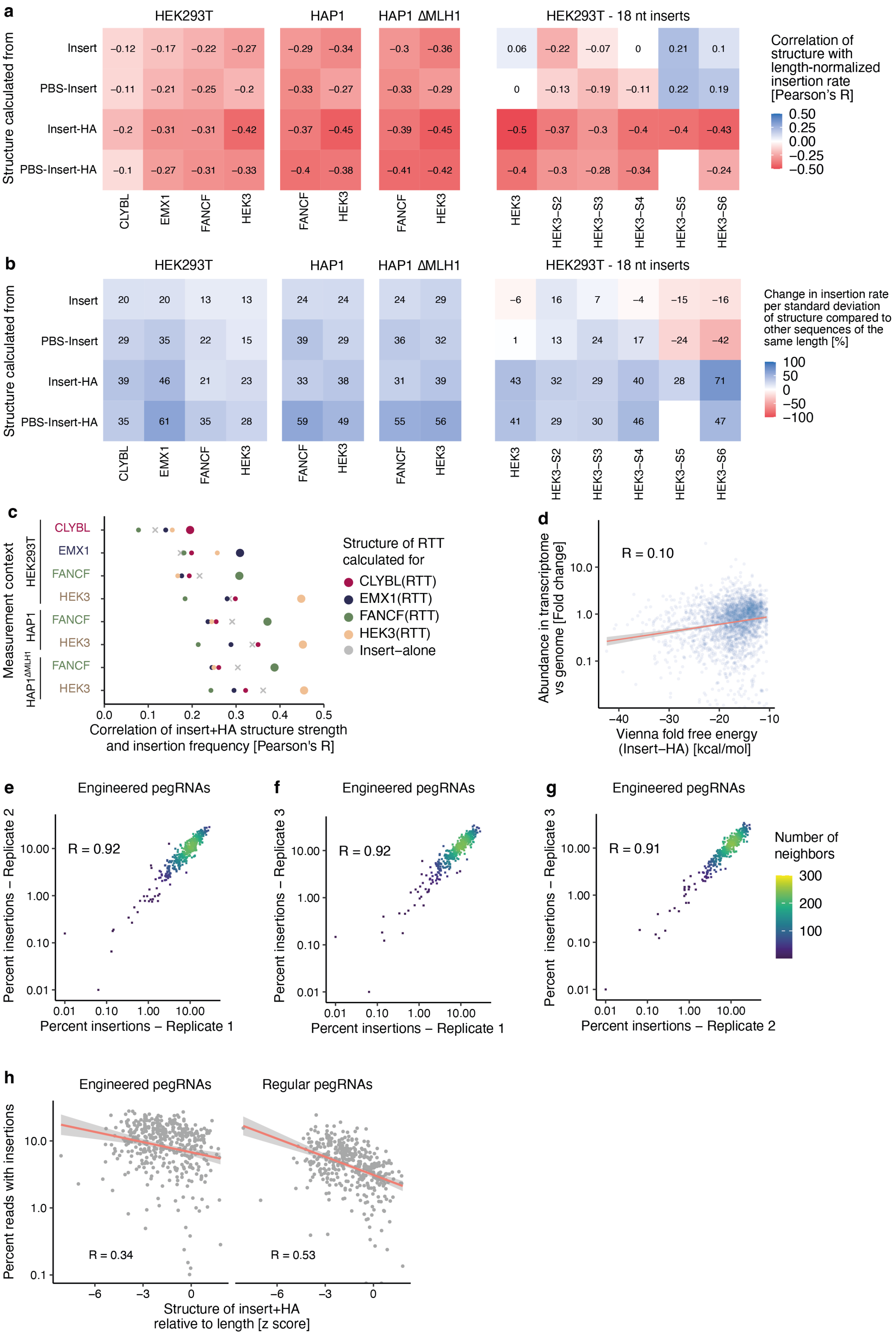
a
HEK293T
HAP1
HAP1 ΔMLH1
HEK293T - 18 nt inserts
Structure calculated from
Insert
PBS-Insert
Insert-HA
PBS-Insert-HA
−0.12 −0.17 −0.22 −0.27 −0.29 −0.34 −0.3 −0.36 0.06 −0.22 −0.07 0 0.21 0.1
−0.11 −0.21 −0.25 −0.2 −0.33 −0.27 −0.33 −0.29 0 −0.13 −0.19 −0.11 0.22 0.19
−0.2 −0.31 −0.31 −0.42 −0.37 −0.45 −0.39 −0.45 −0.5 −0.37 −0.3 −0.4 −0.4 −0.43
−0.1 −0.27 −0.31 −0.33 −0.4 −0.38 −0.41 −0.42 −0.4 −0.3 −0.28 −0.34 −0.24
CLYBL EMX1 FANCF HEK3 FANCF HEK3 FANCF HEK3 HEK3 HEK3−S2 HEK3−S3 HEK3−S4 HEK3−S5 HEK3−S6
Correlation of structure with length-normalized insertion rate [Pearson's R]
0.50 0.25 0.00 −0.25 −0.50
b
HEK293T
HAP1
HAP1 ΔMLH1
HEK293T - 18 nt inserts
Structure calculated from
Insert
PBS-Insert
Insert-HA
PBS-Insert-HA
20 20 13 13 24 24 24 29 −6 16 7 −4 −15 −16
29 35 22 15 39 29 36 32 1 13 24 17 −24 −42
39 46 21 23 33 38 31 39 43 32 29 40 28 71
35 61 35 28 59 49 55 56 41 29 30 46 47
CLYBL EMX1 FANCF HEK3 FANCF HEK3 FANCF HEK3 HEK3 HEK3−S2 HEK3−S3 HEK3−S4 HEK3−S5 HEK3−S6
Change in insertion rate per standard deviation of structure compared to other sequences of the same length [%]
100 50 0 −50 −100
c
Measurement context
HEK293T
HAP1
HAP1ΔMLH1
CLYBL
EMX1
FANCF
HEK3
FANCF
HEK3
FANCF
HEK3
0.0 0.1 0.2 0.3 0.4 0.5
Correlation of insert+HA structure strength and insertion frequency [Pearson's R]
Structure of RTT calculated for
CLYBL(RTT)
EMX1(RTT)
FANCF(RTT)
HEK3(RTT)
Insert−alone
d
R = 0.10
Abundance in transcriptome vs genome [Fold change]
10.0 1.0 0.1
−40 −30 −20 −10
Vienna fold free energy (Insert−HA) [kcal/mol]
e
Engineered pegRNAs
R = 0.92
Percent insertions − Replicate 2
Percent insertions − Replicate 1
0.01 0.10 1.00 10.00
f
Engineered pegRNAs
R = 0.92
Percent insertions − Replicate 3
Percent insertions − Replicate 1
0.01 0.10 1.00 10.00
g
Engineered pegRNAs
R = 0.91
Percent insertions − Replicate 3
Percent insertions − Replicate 2
0.01 0.10 1.00 10.00
Number of neighbors
300 200 100
h
Engineered pegRNAs
Regular pegRNAs
Percent reads with insertions
10.0 1.0 0.1
R = 0.34
R = 0.53
−6 −3 0
Structure of insert+HA relative to length [z score]

Supplementary Figure 8. pegRNA structure affects insertion efficiencies. a. Correlation of length normalized insertion rate (colors) with structure calculated from different parts of the extension (y-axis) in each screen (x-axis, grouped by cell line and screen sets). Data represent the average of *n*=3 (HEK293T) or *n*=2 (HAP1) biological replicates. **b.** Change in length-normalized insertion rate (color) per extra standard deviation of structure calculated from different parts of the extension (y-axis) across different cell lines and targets (x-axis). Data represent the average of *n*=3 (HEK293T) or *n*=2 (HAP1) biological replicates. **c.** Correlation (x-axis) between insertion efficiency in different contexts (y-axis) and pegRNA 3' extension structure free energy calculated for pegRNAs against different target sites (colored markers), or the insert sequence alone (gray cross). **d.** Fold-change at the HEK3 site in HEK293T cells between read counts in the transcriptome and the genome for inserts (markers) with calculated Gibbs free energy (ΔG) from ViennaFold (x-axis). Line: linear regression fit; shaded area: 95% posterior confidence interval of the fit. Data represent the average of n=3 biological replicates. **e.** Percent insertion in replicate 1 (x-axis) compared to percent insertion in replicate 2 for engineered pegRNAs encoding 379 structured inserts (markers) in the HEK293T cell line. **f-g.** As in (e) but for different replicate comparisons. **h.** Insertion rates at the HEK3 site in HEK293T cells relative to length bin median (y-axis) for 379 structured inserts (markers) with calculated Gibbs free energy (ΔG) from ViennaFold (x-axis) stratified by engineered and regular pegRNAs. Line: linear regression fit; shaded area: 95% posterior confidence interval of the fit. Data represent the average of *n*=3 biological replicates.

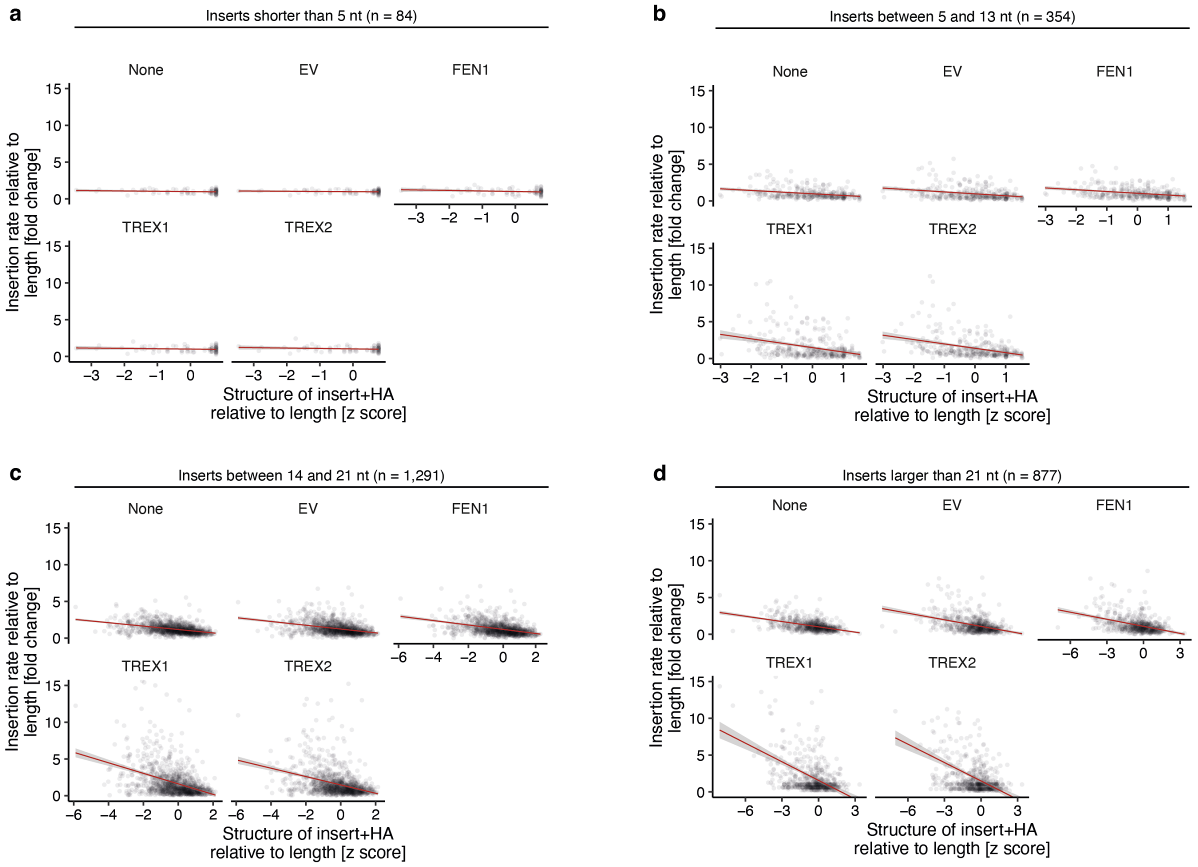

Supplementary Figure 9. Longer sequences benefit more from the additional structure. a. Correlation of insertion rates at the HEK3 site in HEK293T cells relative to length bin median (y-axis) for 84 inserts < 5 nt (markers) with z scores of calculated Gibbs free energy (ΔG) from ViennaFold (x-axis) relative to a large sample of random sequences of the same length. Stratified by overexpression constructs (panels). **b-d.** As (a) but for sequences of different lengths.

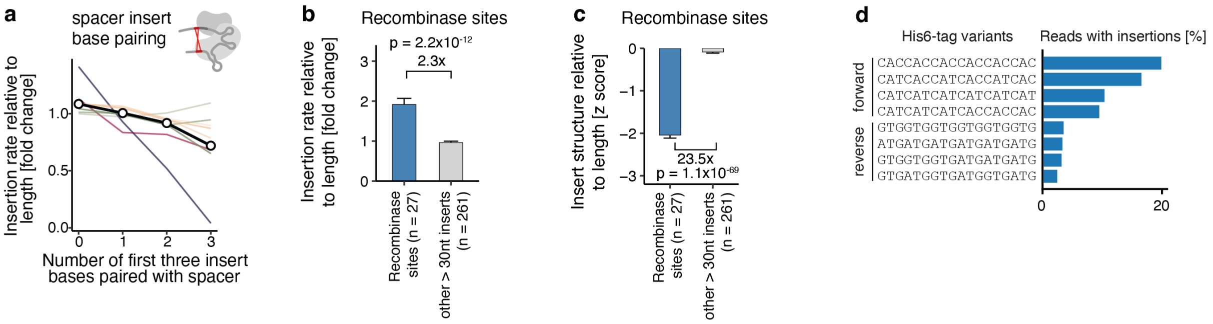

Supplementary Figure 10. Structure and cytosine content explain why some sequences are inserted better than others. a. Insertion rates relative to length bin median (y-axis) for sequences with a different number of bases of the protospacer pairing to the first three nucleotides of the insert (x-axis). Colored lines show screen medians and the thicker black lines and dots show the median across all screens. **b.** Recombinase sites are efficiently inserted relative to their size. Average insertion rate relative to the length bin (y-axis) for recombinase sites or other insert sequences larger than 30 nt (x-axis). Bars: Median and standard error of median Comparison: ratio of blue to grey bar height. p-value: 1.1×10^{-69}; two-sided Student's t-test. n=3 biological replicates. **c.** As (b), but for the secondary structure of the inserts. **d.** The average percent of reads with insertions (x-axis) for different codon versions of the His6 tag in forward and reverse orientation (y-axis) at the HEK3 site in HEK293T cells.

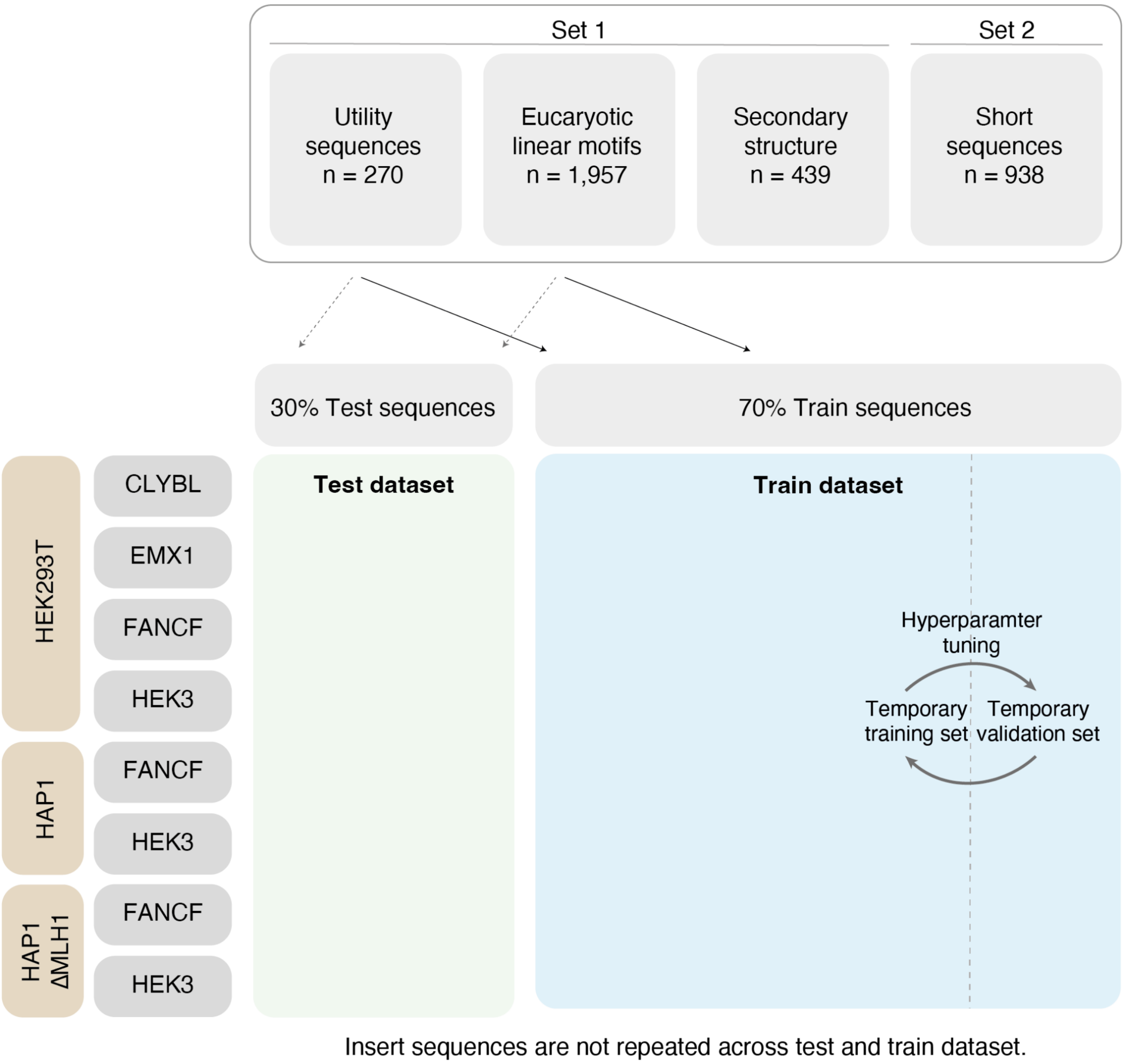

Supplementary Figure 11. Data splitting into test and train sets. The input data of the model consists of sequences from Set 1 and Set 2. The dataset was split into the train (70%) and test (30%) datasets by randomly splitting sequences. To avoid repeated sequences across the test and train datasets, the data points were assigned to the datasets based on the assignment of unique test and train sequences. The hyperparameters of the model were tuned with the train dataset alone.

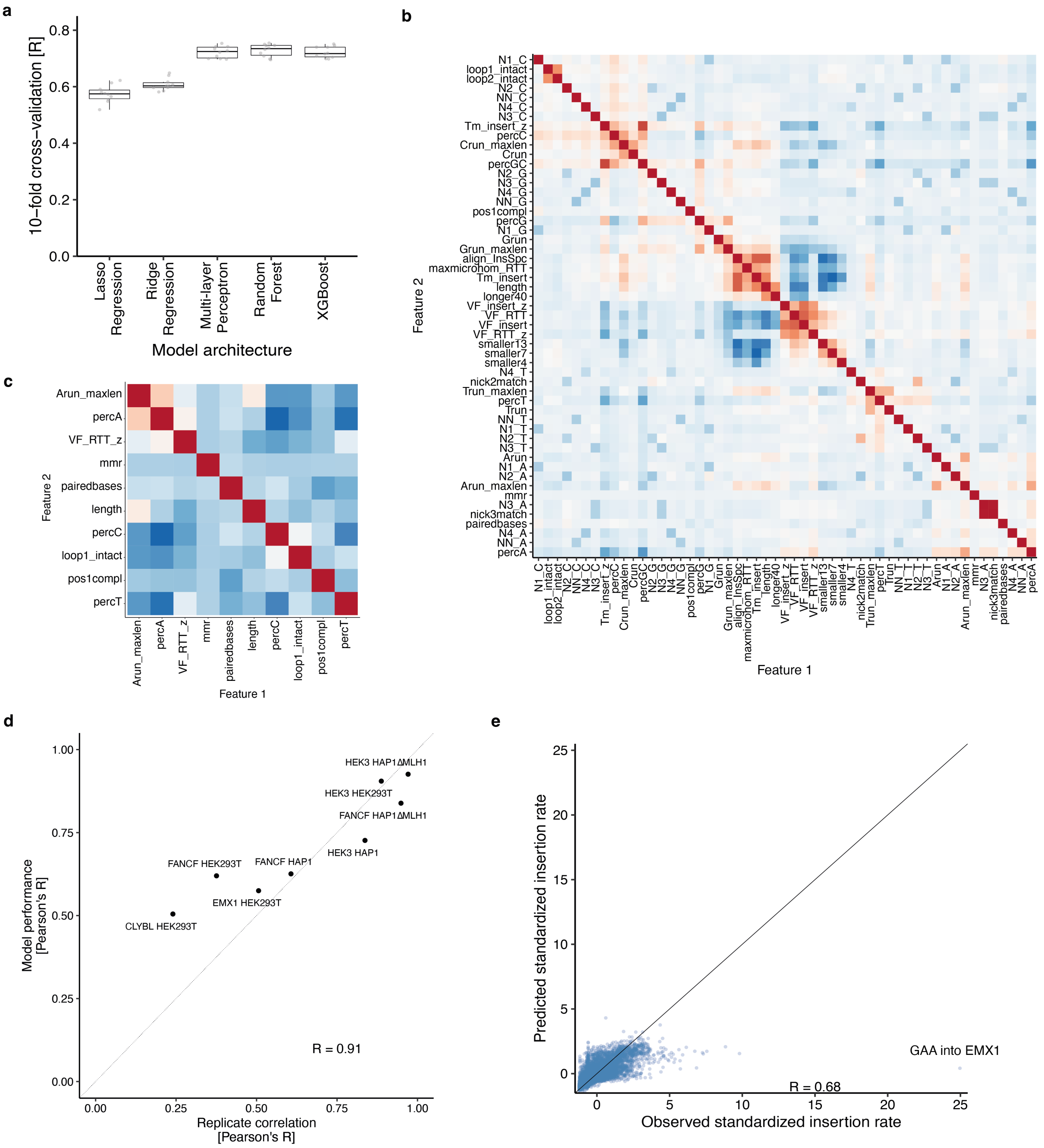

Supplementary Figure 12. Model architectures and features. a. Cross-validation performance of different regression models. Pearson's R between predicted and observed normalized insertion rates (y-axis) for ten cross-validation folds of the training set across a range of models (x-axis) after hyperparameter tuning. Box: median and quartiles; whiskers: least extreme of 1.5 times the interquartile range from the quartile and most extreme values. Observed data for *n*=2 (HAP1) or *n*=3 biological replicates (HEK293T). **b**. Feature correlations. Pearson's correlation (color) of all extracted features (x-axis) with all extracted features (y-axis). **c**. Correlation of model features. Pearson's correlation (color) of all features used in the final model (x-axis) with all features used in the final model (y-axis). **d.** Concordance (Pearson's R) of model performance (y-axis) and replicate correlation (x-axis). Dashed line: y=x. **e.** Concordance of predicted (y-axis) and observed (x-axis) insertion efficiencies on the held-out test set (markers). Solid line: y=x. Label: Pearson's R.

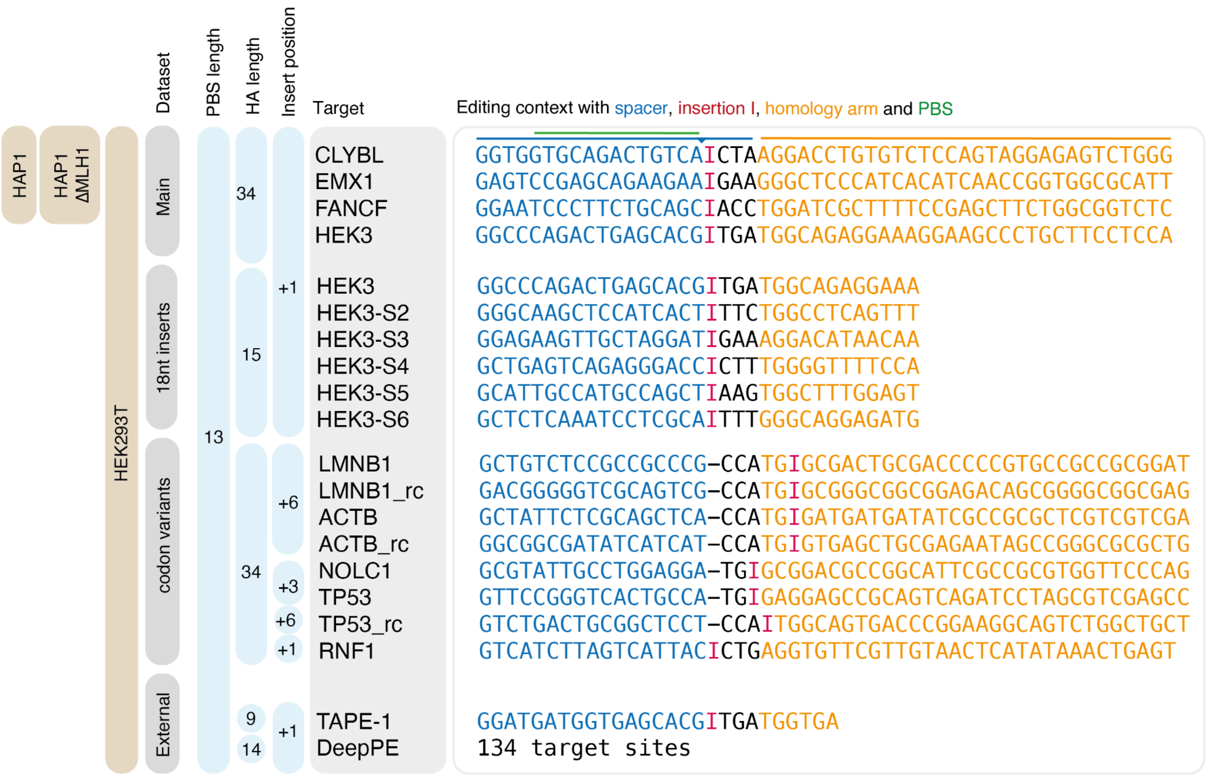

Supplementary Figure 13. Editing contexts for all datasets. For each target site, spacer (blue), homology sequences (orange), insert position (red), and pegRNA properties, including PBS length, homology arm length, and insertion position are indicated.

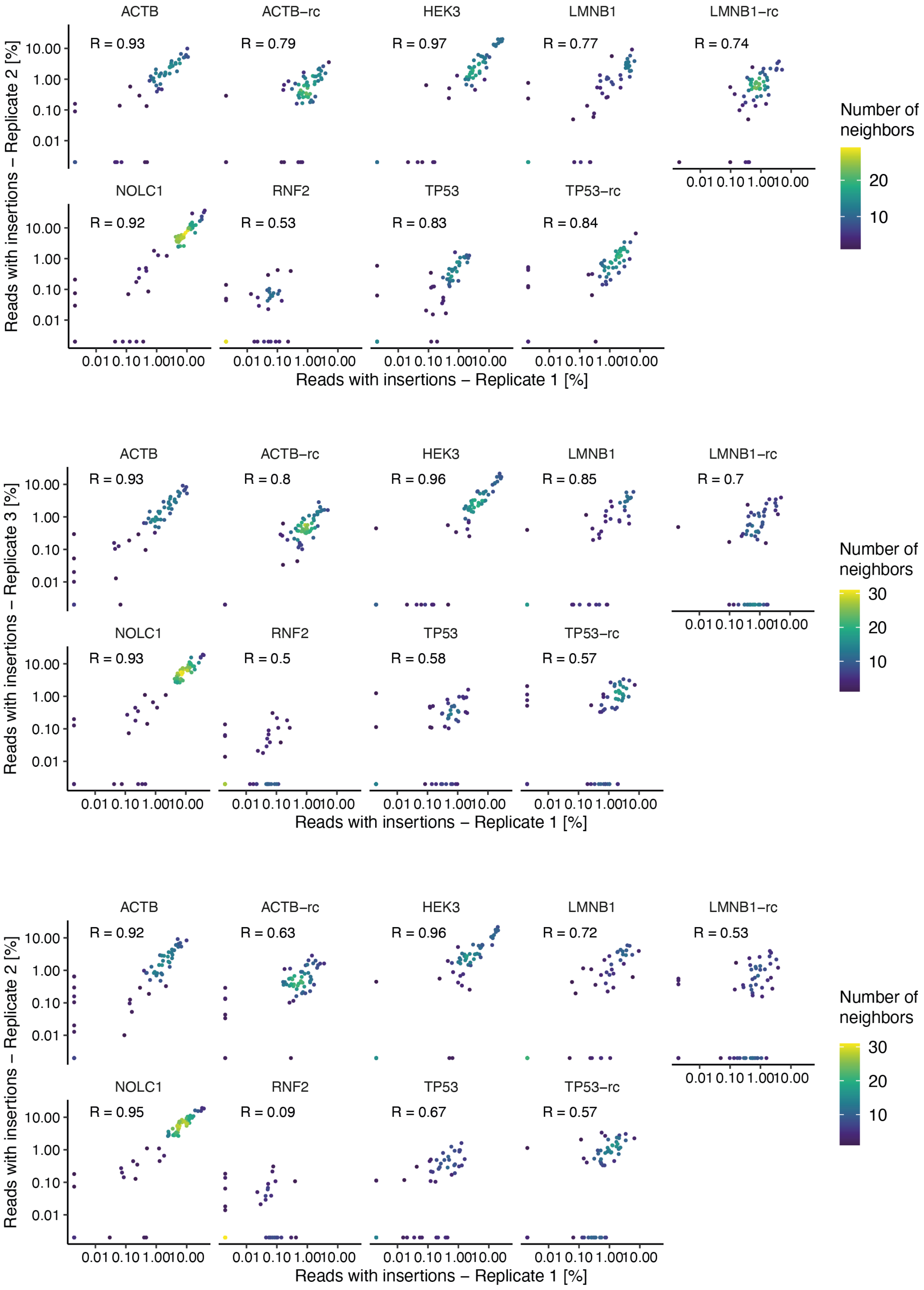

Supplementary Figure 14. Replicate correlation for insertions of protein tags into new targets. Percent insertion in one replicate (x-axis) compared to percent insertion in another replicate for the new set of screens with protein tag sequences (markers) at different nine target sites (rows) in HEK293T cells.

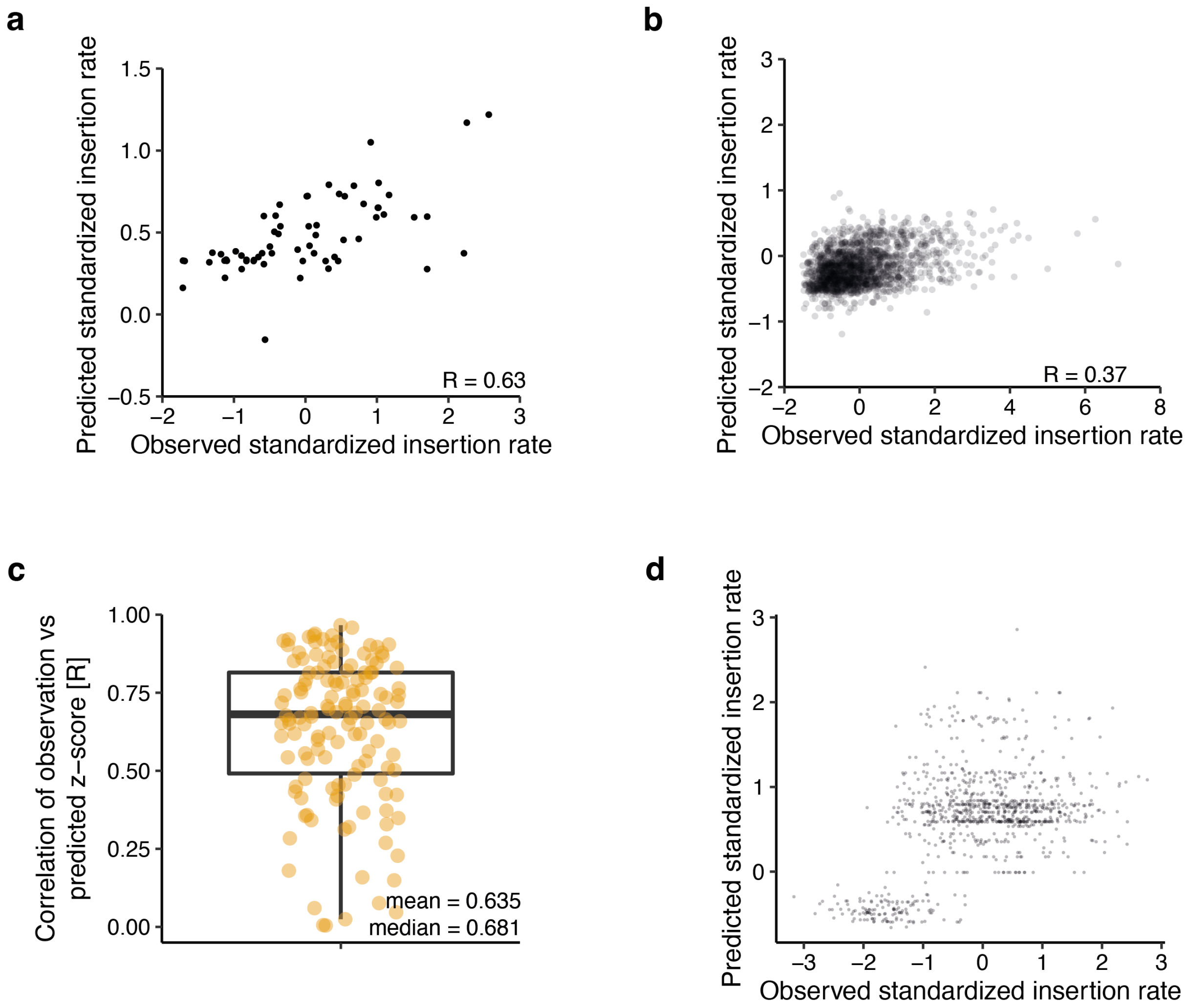

Supplementary Figure 15. Validation of MinsePIE on external datasets. a. Concordance of observed and predicted values for 6 nt insertions from Choi et al. **b.** as a, but for 9 nt insertions. **c.** Concordance of observed and predicted relative insertion rates for 134 target sites from Kim et al. per target site. Box: median and quartiles; whiskers: least extreme of 1.5 times the interquartile range from the quartile and most extreme values. **d.** Concordance of predicted (y-axis) and observed (x-axis) insertion efficiencies for all sequences in the Kim et al. data set (markers). Solid line: y=x. Label: Pearson's R.

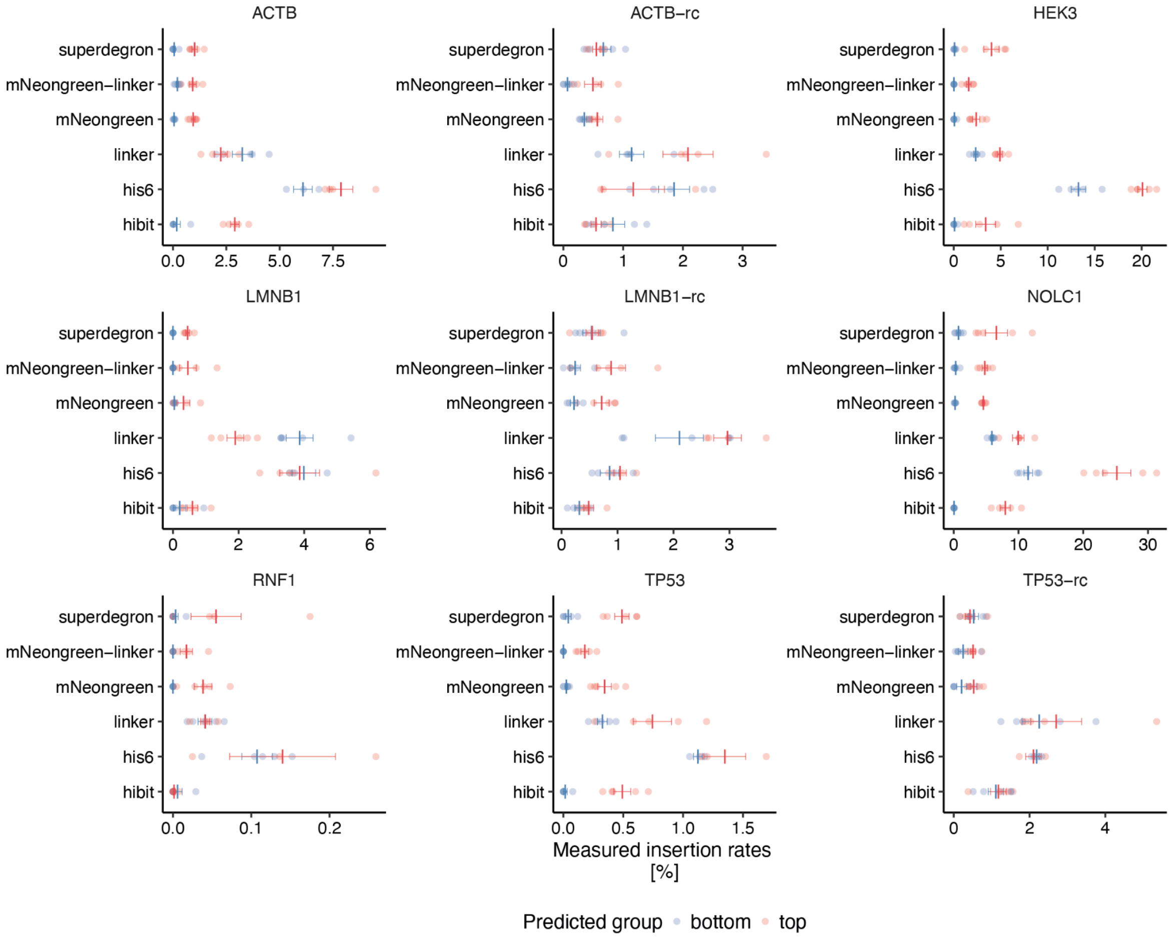

Supplementary Figure 16. Predicting high- and low-inserting codon versions of protein tags. MinsePIE was used to predict insertion efficiencies for generated codon variants to select 66 codon variants of six protein tags (superdegron, mNeongreen11-linker, mNeongreen-11, glycine-rich linker, His-6, and HiBiT) to generate in-frame fusions for nine target sites in HEK293T cells with either high or low efficiency. Insertion rate (x-axis) for codon variants (markers) of six protein tags (y-axis). Red: high predicted insertion rate; blue: low predicted insertion rate. Bar and whiskers: mean and standard error of the mean.

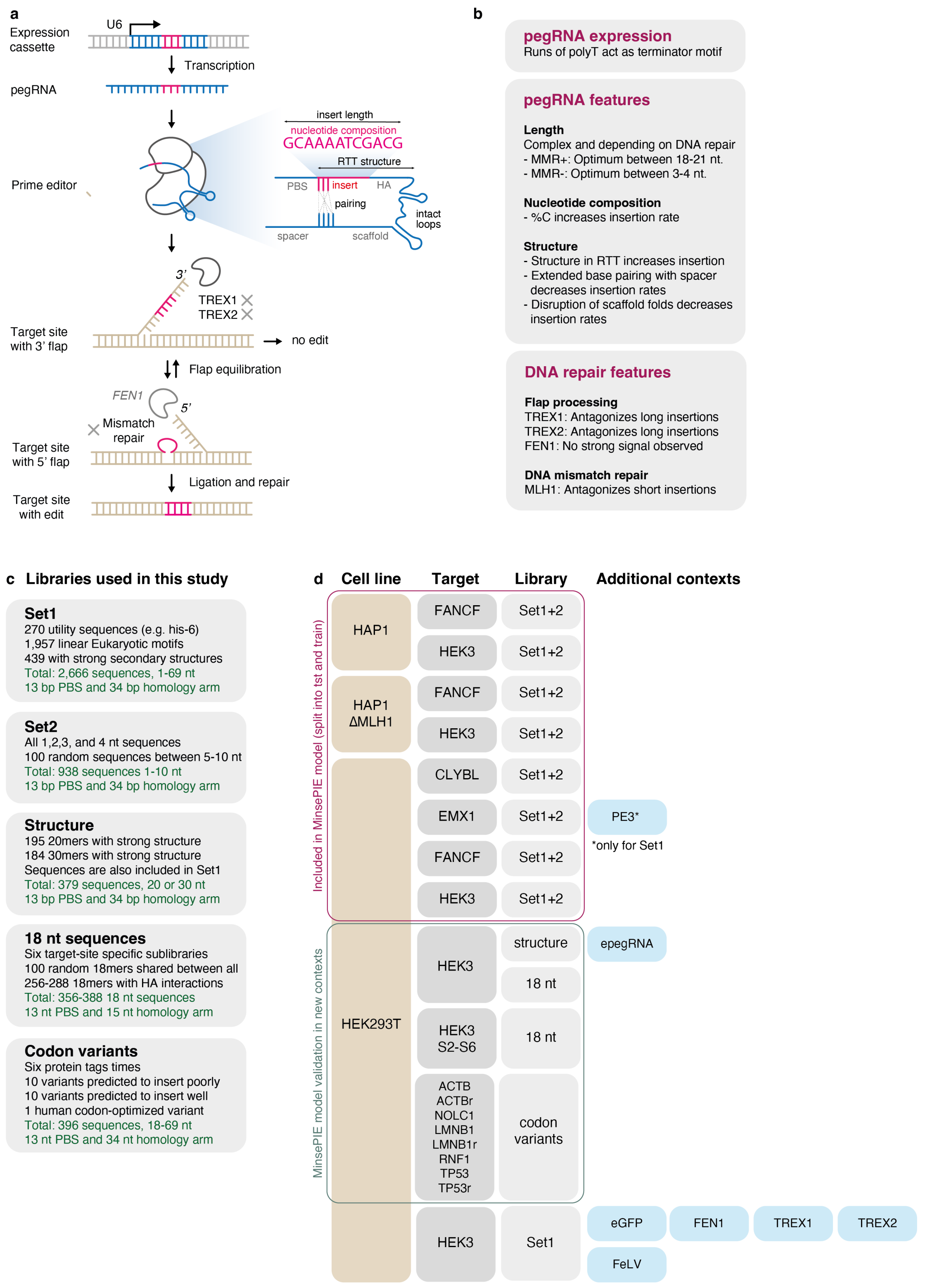

Supplementary Figure 17. Overview of the pathway, features, libraries, and screens explored in this study. a. Schematic of molecular steps involved in prime editing. The steps addressed in this manuscript are indicated and important features that affect prime insertion efficiencies are shown. **b.** Collection of pegRNA and DNA repair features and their effects on insertions. **c.** Collection of the libraries used in this study. **d.** Overview of the screens performed in this study, showing the cell lines, target sites, libraries, and additional contexts tested.

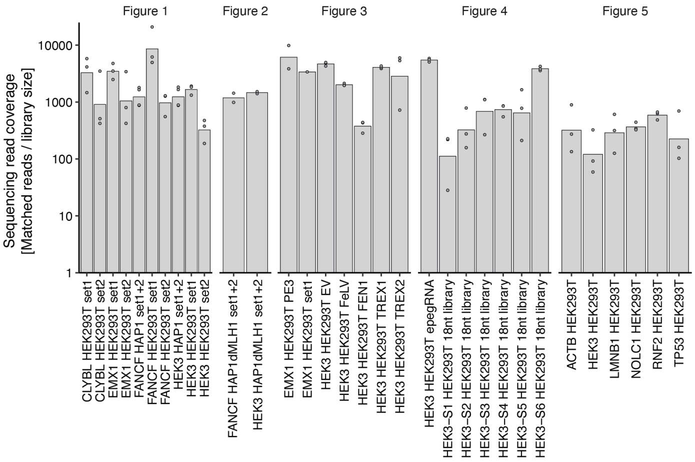

Supplementary Figure 18. Sequencing coverage for all screens. Sequencing coverage (raw sequencing read counts for reads that matched a library insert or the wild type sequence divided by the size of the screened library - y-axis) for all screens (x-axis) stratified by the figures in which they first appear (panels).

Supplementary Note 1: Codon choice for insertion sequences

As many factors determine the insertion efficiencies we generally recommend using MinsePIE to predict insertion efficiency for all possible codon variants (https://elixir.ut.ee/minsepie/ or https://github.com/julianeweller/MinsePIE). For a quick glance at the insertion efficiencies of codons, we decomposed all sequences that were divisible by three into individual codons and tracked the association of codon usage with editing rate. Codon preference generally reflected cytosine content. Efficient codons had at least two Cs (6 out of the top 10 preferred codons). Poorly performing codons conversely are AT-rich. The AAA codon (lysine) performs notably worse than all other codons, presumably because of its potential to form polymerase III stop signals. Runs of four or more consecutive adenines should be avoided if possible as they are highly detrimental to editing efficiencies. If adenine runs cannot be avoided, another possibility is to target the reverse strand if compatible PAM sites are available.

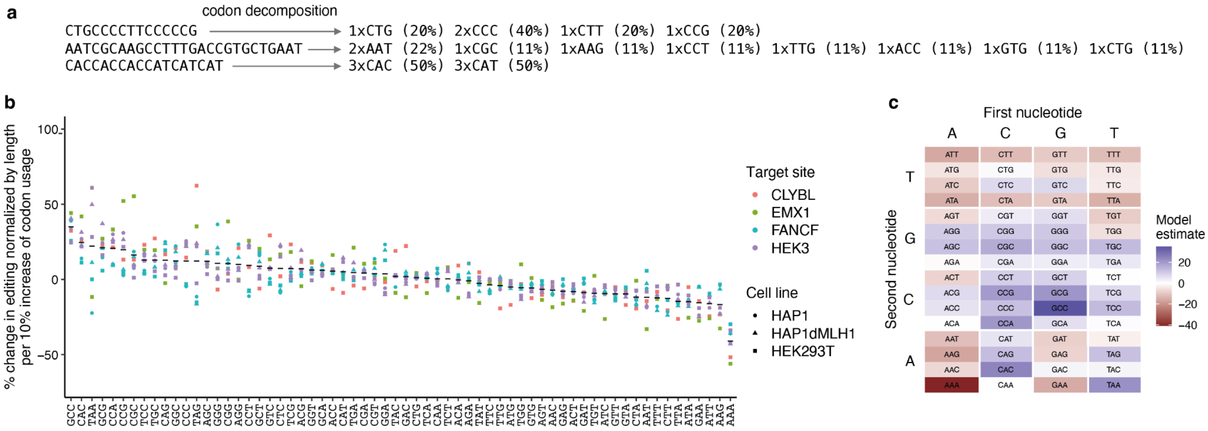

Codon usage table. **a.** Codon decomposition for insert sequences in the library that are divisible by three. Each sequence is encoded by the percent of each codon it is made up of. **b.** Influence of codon usage (x-axis) on editing rates relative to length bin median (y-axis) for individual screens (markers) stratified by target (color) and cell lines (shape). Crossbars represent the mean. Data represent the average of n=3 biological replicates. **c.** Percent additional insertion relative to the average length-normalized insertion rate (color) per extra ten percent of codon usage in the insert sequence. Arranged by the first (x-axis) and second (y-axis) nucleotide of the codon. Data represent the average of n=3 biological replicates.

Supplementary Table 1. Explanation of feature sets used in the MinsePIE model with an explanation of how they were calculated, example plots, correlation with insertion rates or length normalized insertion rates in a linear model with only the given feature, mean absolute SHAP value in the MinsePIE model, and generally good feature values.

**Feature**	**Description and how the feature was calculated**	**Example plot**	**Median correlation in linear model**	**Mean absolute shap value**	**Good values for insertion**
Length	Length of the insert sequence<br><br>Value range: 1-69 nt	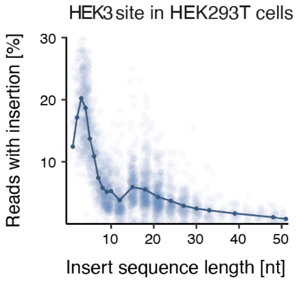	R = 0.34<br>(~ %insertion)	0.37	*MMR positive:*<br>Optimum: 18-21nt<br><br>*MMR negative:*<br>Optimum: 3-4nt<br>2nd optimum: 18-21nt
% adenine<br>% cytosine<br>% thymine	Nucleotide content of the insert sequence (n Ns/n nucleotides)*100<br><br>Value range: 0-100 %	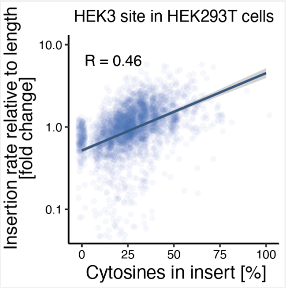	for % C:<br>R = 0.23<br>(~ length normalized %insertion)	0.04 (%A)<br>0.14 (%C)<br>0.02 (%T)	Optimal: High cytosine content
structure insert-HA	The structure of the insert sequence and homology arm compared to the structure of 1000 random sequences<br><br>Value range: -8.1-3.4	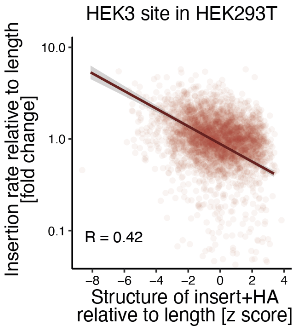	R = -0.38<br>(~ length normalized %insertion)	0.16	Optimal: Strong structure in insert and HA
Runs of adenines	The longest consecutive run of adenines in the insert sequence in the context of the two nucleotides flanking it<br><br>Value range: 1-6+	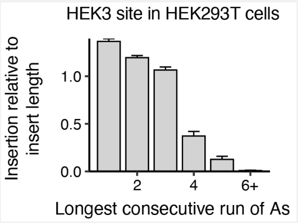	R = -0.26<br>(~ length normalized %insertion)	0.05	Optimal: No runs of 4 or more adenines.

<table>
<tr><td>Paired bases with PBS</td><td>Pairing of the first insert bases with positions 17-20 on the spacer and position 1 on scaffold

Value range: 0-3+</td><td>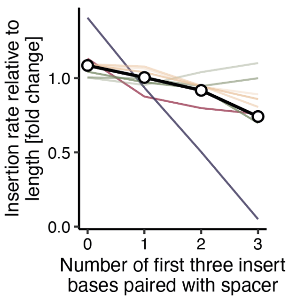
</td><td>R = -0.07
(~ length normalized %insertion)</td><td>0.05</td><td>Optimal: 0 bases paired with PBS</td></tr>
<tr><td>Pairing nt1:spacer</td><td>Pairing of the first insert base with position 17 on the spacer

Value range: False, True</td><td>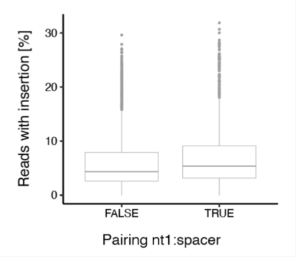
</td><td>R = 0.06
(~ %insertion)</td><td>0.02</td><td></td></tr>
<tr><td>Scaffold loop1 intact</td><td>Correct folding of the first scaffold loop (positions 1-30)

The structure was calculated for the scaffold pasted together with “NNN” and the reverse complement of the insert sequence using Vienna fold. Loop 1 was intact if positions 1-30 were "(((((((.((((....))))...)))))))"

Value range: 0 (disrupted), 1 (intact)</td><td>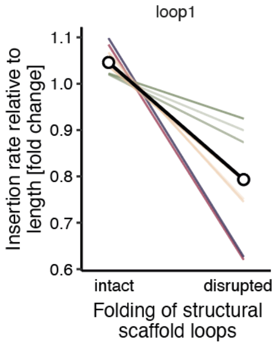
</td><td>R = -0.1
(~ length normalized %insertion)</td><td>0.001</td><td>Optimal: Inserts that do not disrupt the scaffold</td></tr>
<tr><td>Mismatch repair</td><td>Mismatch repair status of the cell line (proficient: HAP1, deficient: HAP1 ∆MLH1, HEK293T)

Value range: 0 (deficient), 1 (proficient)</td><td>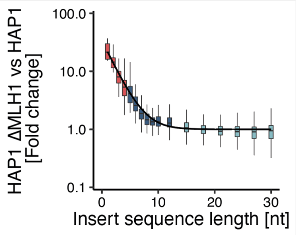
</td><td>NA</td><td>0.09</td><td>MMR-deficient cell lines work better for inserts < 13 nt</td></tr>
</table>

Correlations and other measures of feature importance were calculated with a data set that contained screens for the CLYBL, EMX1, FANCF, HEK3 sites in HEK293T cells and the FANCF and HEK3 sites in HAP1 and HAP1 ∆MLH1 cells.

Supplementary Table 2. Feature sets used for evaluation of prediction models (Figure 5)

Feature set	Figure	Features
Model	5	Length, normalized free energy of the RT template, MMR, %C, paired bases insert:(spacer+G), maximum length of A run, %A, %T, pairing of 1st insert nucleotide and spacer, loop 1 intact
System	5b	MMR, the maximum length of A run
Length	5b	Length
Structure	5b	normalized free energy of the RT template, paired bases insert:(spacer+G), loop 1 intact, pairing of 1st insert nucleotide and spacer
%N	5b	%C, %A, %T
Sequence	5b	Length, normalized free energy of the RT template, paired bases insert:(spacer+G), loop 1 intact, pairing of 1st insert nucleotide and spacer, %C, %A, %T
Extra	5b	Length, normalized free energy of the RT template, free energy of the insert, normalized free energy of the insert, melting temperature of the insert, normalized melting temperature of the insert, paired bases insert:(spacer+G), loop 1 intact, loop 2 intact, pairing of 1st insert nucleotide and spacer, pairing of 2nd insert nucleotide and spacer, pairing of 3rd insert nucleotide and spacer, alignment score between insert and spacer, microhomology of RT template, %C, %A, %T, %G, %GC, smaller than 4nt, smaller than 7nt, smaller than 13nt, longer than 40nt, MMR, maximum length of A run, maximum length of T run, maximum length of G run, maximum length of C run, C run, G run, A run, T run, N1 = A, N2 = A, N3 = A, N4 = A, last N = A, N1 = T, N2 = T, N3 = T, N4 = T, last N = T, N1 = C, N2 = C, N3 = C, N4 = C, last N = C, N1 = G, N2 = G, N3 = G, N4 = G, last N = G

Supplementary Table 3. Sequences of oligonucleotides used in this study

ID	Name	Sequence	Purpose
P1	CLYBL_S2_F	ACACTCTTTCCCTACACGACGCTCTTCCGATCTGAAGACCCCAGTGATTCATGCCTC	NGS of CLYBL target site
P2	CLYBL_S4_F	ACACTCTTTCCCTACACGACGCTCTTCCGATCTACGAAGACCCCAGTGATTCATGCCTC	NGS of CLYBL target site
P3	CLYBL_iPCR_R	GAGATCGGTCTCGGCATTCCTGCTGAACCGCTCTTCCGATCTGGCTTGACTAGGGCTGGATGAT	NGS of CLYBL target site
P4	1114_EMX1_S1_F	ACACTCTTTCCCTACACGACGCTCTTCCGATCTACAGCTCAGCCTGAGTGTTGA	NGS of EMX1 target site
P5	1115_EMX1_S7_F	ACACTCTTTCCCTACACGACGCTCTTCCGATCTGACGACACAGCTCAGCCTGAGTGTTGA	NGS of EMX1 target site
P6	1116_EMX1_ R	GAGATCGGTCTCGGCATTCCTGCTGAACCGCTCTTCCGATCTCTCGTGGGTTTGTGGTTGC	NGS of EMX1 target site
P7	912_NGS_HEK3_F_S0	ACACTCTTTCCCTACACGACGCTCTTCCGATCTATGTGGGCTGCCTAGAAAGG	NGS of HEK3 target site
P8	913_NGS_HEK3_F_S8	ACACTCTTTCCCTACACGACGCTCTTCCGATCTTGGACACAATGTGGGCTGCCTAGAAAGG	NGS of HEK3 target site
P9	995_NGS_HEK3 _R	GAGATCGGTCTCGGCATTCCTGCTGAACCGCTCTTCCGATCTCCCAGCCAAACTTGTCAACC	NGS of HEK3 target site
P10	1000_FANCF_F_S3	ACACTCTTTCCCTACACGACGCTCTTCCGATCTCGAattgcagagaggcgtatca	NGS of FANCF target site
P11	1001_FANCF_F_S6	ACACTCTTTCCCTACACGACGCTCTTCCGATCTCTAGAAattgcagagaggcgtatca	NGS of FANCF target site
P12	1002_FANCF_R	GAGATCGGTCTCGGCATTCCTGCTGAACCGCTCTTCCGATCTGGGGTCCCAGGTGCTGAC	NGS of FANCF target site
P13	962_Seqlib_S2_F	ACACTCTTTCCCTACACGACGCTCTTCCGATCTGATGGCTTTATATATCTTGTGGAAAGGACGAAACACC	NGS of pegRNA library
P14	963_Seqlib_S6_F	ACACTCTTTCCCTACACGACGCTCTTCCGATCTCTAGAATGGCTTTATATATCTTGTGGAAAGGACGAAACACC	NGS of pegRNA library
P15	965_P7_Broad_R	GTGACTGGAGTTCAGACGTGTGCTCTTCCGATCTTCTACTATTCTTTCCCCTGCACTGT	NGS of pegRNA library
P16	Goose_F_Universal	TTAAGCAAGCAAGCGAGCACTC	Amplification of oligos from pool
P17	Goose_CLYBL_R	GCGTCAATTCAGGCAACGAAGA	Amplification of oligos from pool
P18	Goose_EMX1_R	GCTTCAAGCCCTAGTGTCCTCA	Amplification of oligos from pool
P19	Goose_FANCF_R	ACAACTCTCTGAATGCGCTTGC	Amplification of oligos from pool
P20	Goose_HEK3_R	CCCGAGGAAATGATAGGGCGAT	Amplification of oligos from pool
P21	PE1.0	AATGATACGGCGACCACCGAGATCTACACTCTTTCCCTACACGACGCTCTTCCGATC*T	Forward primer indexing PCR
P22	Rev primer	CAAGCAGAAGACGGCATACGAGATN10GAGATCGGTCTCGGCATTCCTGCTGAACCGCTCTTCCGATCT	Reverse primer for indexing PCR
P23	ACTB_NGS_F	ACACTCTTTCCCTACACGACGCTCTTCCGATCTCGACCTCGGCTCACAGCG	NGS of ACTB target site
P24	ACTB_NGS_R	GAGATCGGTCTCGGCATTCCTGCTGAACCGCTCTTCCGATCTCCACCCAGCCAGCTCCC	NGS of ACTB target site
P25	LMNB1_NGS_F	ACACTCTTTCCCTACACGACGCTCTTCCGATCTCGCTTCGCCCCCTGCC	NGS of LMNB1 target site
P26	LMNB1_NGF_R	GAGATCGGTCTCGGCATTCCTGCTGAACCGCTCTTCCGATCTGGTCATTGAGCTCGCGCAGC	NGS of LMNB1 target site
P27	NOLC1_NGS_F	ACACTCTTTCCCTACACGACGCTCTTCCGATCTGAGTCGTGCTGCGTCGACAA	NGS of NOLC1 target site
P28	NOLC1_NGS_R	GAGATCGGTCTCGGCATTCCTGCTGAACCGCTCTTCCGATCTTGGCGAACTTATTGGCCACCTC	NGS of NOLC1 target site
P29	RNF2_NGS_F	ACACTCTTTCCCTACACGACGCTCTTCCGATCTACGTCTCATATGCCCCTTGG	NGS of RNF2 target site
P30	RNF2_NGS_R	GAGATCGGTCTCGGCATTCCTGCTGAACCGCTCTTCCGATCTACGTAGGAATTTTGGTGGGACA	NGS of RNF2 target site
P31	TP53_NGS_F	ACACTCTTTCCCTACACGACGCTCTTCCGATCTgctggatccccacttttcctct	NGS of TP53 target site
P32	TP53_NGS_R	GAGATCGGTCTCGGCATTCCTGCTGAACCGCTCTTCCGATCTttttcgcttcccacaggtctct	NGS of TP53 target site

Supplementary Table 4. Plasmids used in this study

Name	Description	Benchling link
pCMV-PE2-P2A-PuroR	Prime editor expression plasmid with puromycin resistance	https://benchling.com/s/seq-JxYVGybwwOovqgONpITH?m=slm-7R0qOn9t8xIHTPi7UZno
pLentiGuide-BlastR	Lentiviral acceptor vector for pegRNAs with blasticidin resistance	https://benchling.com/s/seq-of3MsHcYymrO04VXMqN5?m=slm-6njEl8yUYq48oeEWe8nG
pLentiGuide-BlastR-Library	Example of a library vector containing the loxP site and targeting the FANCF locus	https://benchling.com/s/seq-FjvxjpC95r4xbyJUBhQd?m=slm-iW7NNuOXt9FzJyXv5YBb
pPB-TREG3G-PE2-rtTA3G-P2A-eGFP	Piggybac vector with doxycyclin-inducible prime editor	https://benchling.com/s/seq-rCcJG0pk2TUvOSVljikl?m=slm-2LxVK7M5LvfREDcBRfgX
pTwist_EF1a_FEN1-T2A-tagBFP	FEN1 and tBFP overexpression vector	https://benchling.com/s/seq-P9kog1NtZ4NGlP84RcPL?m=slm-uxtWyuiTq9hk2EKYig0o
pTwist_EF1a_TREX1-T2A-mScarlet	TREX1 and mScarlet overexpression vector	https://benchling.com/s/seq-bDzcTrQqGtapgEJDOxLy?m=slm-VuDGiBXtGTrXBejWriiA
pTwist_EF1a_TREX2-T2A-emiRFP670	TREX2 and emiRFP670 overexpression vector	https://benchling.com/s/seq-fE0LXpErRwfbgEGF5frx?m=slm-h6ggd0mB9n7BCDbjVYMK
pTwist_EF1a_Acceptor-T2A-eGFP	Overexpression vector with a cloning site and eGFP	https://benchling.com/s/seq-C6ZKV8n8oCzml5oufiuw?m=slm-Mt6rXWRjdQZvWbdVrmgw

Supplementary Tables 5 and 6 are provided in a separate Microsoft Excel file.

Supplementary Table 5. Sequences of all gene fragments ordered

Supplementary Table 6. Sequences of pegRNA libraries and individual pegRNAs used in this study.
