# Supplementary material for: Prediction of prime editing insertion efficiencies using sequence features and DNA repair determinants
Source: Nat Biotechnol. 2023 Feb 16;41(10):1446–56. doi: 10.1038/s41587-023-01678-y (PMC10567557; doi:10.1038/s41587-023-01678-y)
Supplement: Supplementary file 2 — Reporting Summary [file 41587_2023_1678_MOESM2_ESM.pdf]

nature portfolio

Corresponding author(s): Leopold Parts

Last updated by author(s): Jan 7, 2023

Reporting Summary

Nature Portfolio wishes to improve the reproducibility of the work that we publish. This form provides structure for consistency and transparency in reporting. For further information on Nature Portfolio policies, see our Editorial Policies and the Editorial Policy Checklist.

Statistics

For all statistical analyses, confirm that the following items are present in the figure legend, table legend, main text, or Methods section.

n/a	Confirmed	
☐	☒	The exact sample size (*n*) for each experimental group/condition, given as a discrete number and unit of measurement
☐	☒	A statement on whether measurements were taken from distinct samples or whether the same sample was measured repeatedly
☐	☒	The statistical test(s) used AND whether they are one- or two-sided<br>*Only common tests should be described solely by name; describe more complex techniques in the Methods section.*
☒	☐	A description of all covariates tested
☐	☒	A description of any assumptions or corrections, such as tests of normality and adjustment for multiple comparisons
☐	☒	A full description of the statistical parameters including central tendency (e.g. means) or other basic estimates (e.g. regression coefficient) AND variation (e.g. standard deviation) or associated estimates of uncertainty (e.g. confidence intervals)
☐	☒	For null hypothesis testing, the test statistic (e.g. *F*, *t*, *r*) with confidence intervals, effect sizes, degrees of freedom and *P* value noted<br>*Give P values as exact values whenever suitable.*
☒	☐	For Bayesian analysis, information on the choice of priors and Markov chain Monte Carlo settings
☒	☐	For hierarchical and complex designs, identification of the appropriate level for tests and full reporting of outcomes
☐	☒	Estimates of effect sizes (e.g. Cohen's *d*, Pearson's *r*), indicating how they were calculated

Our web collection on statistics for biologists contains articles on many of the points above.

Software and code

Policy information about availability of computer code

Data collection	Data was collected on commercial Illumina sequencing machines (Miseq and Hiseq 2500) using the BaseSpaceCLI 1.4.0 software.
Data analysis	PEAR (0.9.11) was used to merge forward and reverse reads. Custom R (4.0.2) and python scripts were then used to process the fastq files and analyze the data (3.8.10). The scripts are deposited at: https://github.com/julianeweller/MinsePIE. The following software was used: BaseSpaceCLI (1.4.0); Geneius codon optimization tool from Eurofins Genomics (accessed 2022); PEAR (0.9.11); Python (3.8.10); Python packages: Biopython (1.79), more-itertools (8.5.0), pandarallel (1.6.1), scikit-learn (0.24.2), scipy (1.5.3), shap (0.39.0), statannot (0.2.3), XGBoost (1.4.0); R (4.0.2); ViennaRNA (2.5.0); R packages: Broom (0.7.9), fuzzyjoin (0.1.6), ggpointdensity (0.1.0), RBioinf (1.48.0), reversetranslate (1.0.0), ShortRead (1.46.0), spgs (1.0-3), Tidyverse (1.3.1), Viridis (0.6.1).

For manuscripts utilizing custom algorithms or software that are central to the research but not yet described in published literature, software must be made available to editors and reviewers. We strongly encourage code deposition in a community repository (e.g. GitHub). See the Nature Portfolio guidelines for submitting code & software for further information.

Data

Policy information about availability of data

All manuscripts must include a data availability statement. This statement should provide the following information, where applicable:

- Accession codes, unique identifiers, or web links for publicly available datasets
- A description of any restrictions on data availability
- For clinical datasets or third party data, please ensure that the statement adheres to our policy

Read count tables for all screens, mutation frequencies at each position and sequences with indels are attached as Supplementary Data files. Figures with associated raw data: Figure 1, Figure 2, Figure 3, Figure 4, Figure 5. Associated with Data_2_insertion_frequencies.

Human research participants

Policy information about studies involving human research participants and Sex and Gender in Research.

Reporting on sex and gender	NA
Population characteristics	NA
Recruitment	NA
Ethics oversight	NA

Note that full information on the approval of the study protocol must also be provided in the manuscript.

Field-specific reporting

Please select the one below that is the best fit for your research. If you are not sure, read the appropriate sections before making your selection.

☒ Life sciences ☐ Behavioural & social sciences ☐ Ecological, evolutionary & environmental sciences

For a reference copy of the document with all sections, see nature.com/documents/nr-reporting-summary-flat.pdf

Life sciences study design

All studies must disclose on these points even when the disclosure is negative.

Sample size	No sample size calculation was performed. Sample sizes were picked to have adequate signal to noise ratio and repeatability in the measurements. The sample size (n) of each experiment is provided in the figure captions. Each experiment was performed in at least two biological replicates. The prime editing screen libraries contained thousands of sequences, providing internal replication.
Data exclusions	No data was excluded from the analysis.
Replication	All experiments were performed with 2 or 3 biological replicates. Biological replicates were independently infected/transfected and kept separate throughout the experiment. For all screens, replicate correlations between all replicate permutations are provided in the Supplementary Figures which include scatter plots for visual inspection and calculated Pearson's R.
Randomization	No randomization was performed. Prime editing screens were performed in a pooled setup which is intrinsically randomized (i.e. all pegRNAs are expressed in random cells depending on which cell was infected with which pegRNA containing lentivirus).
Blinding	Controls and samples were analyzed in exactly the same way using the same computational pipeline. The investigators were blinded to the individual sequences due to the nature of pooled screens, and all library constituents were analyzed.

Reporting for specific materials, systems and methods

We require information from authors about some types of materials, experimental systems and methods used in many studies. Here, indicate whether each material, system or method listed is relevant to your study. If you are not sure if a list item applies to your research, read the appropriate section before selecting a response.

Materials & experimental systems

n/a	Involved in the study
☒	☐ Antibodies
☐	☒ Eukaryotic cell lines
☒	☐ Palaeontology and archaeology
☒	☐ Animals and other organisms
☒	☐ Clinical data
☒	☐ Dual use research of concern

Methods

n/a	Involved in the study
☒	☐ ChIP-seq
☒	☐ Flow cytometry
☒	☐ MRI-based neuroimaging

Eukaryotic cell lines

Policy information about cell lines and Sex and Gender in Research

Cell line source(s)	HEK293T cells were acquired from AMS Bio (EP-CL-0005, Lot 8400B013008). HAP1 (C631) and HAP1 ΔMLH1 cells (HZGHC000343c022) were acquired from Horizon Biosciences.
Authentication	The cell lines were not authenticated. MLH1 knockout (13bp deletion in exon2) was confirmed by DNA Sanger Sequencing.
Mycoplasma contamination	All cell lines tested negative for Mycoplasma
Commonly misidentified lines (See ICLAC register)	None of commonly misidentified lines were used in this study.
